# Drivers of autochthonous malaria cases over time: could the Central European present the African future?

**DOI:** 10.1186/s12936-024-05004-y

**Published:** 2024-06-10

**Authors:** Zoltán Kenyeres

**Affiliations:** Acrida Conservational Research L.P., Deák F. St. 7., Tapolca, 8300 Hungary

**Keywords:** Aerial photographs, Drainage, Marshland, QGIS, Rural, Urban, Nineteenth century

## Abstract

**Background:**

Results of spatial and temporal comparison of malaria hotspots and coldspots could improve the health measures of malaria control and eradication strategies. The study aimed to reveal the spatially and temporally independent correlations between the potentially most effective background variables and the number of autochthonous malaria cases.

**Methods:**

Relationships between malaria cases and background variables were studied in 2 km × 2 km sized quadrates (10 Central European and 10 African). In addition to the current habitat structure of the African sites, annual precipitation, and annual mean temperature, data of the above parameters detected in the nineteenth and twentieth centuries and currently in the Central European sites were included in the analyses (n = 40). Mann–Whitney tests, Principal Component Analysis, and Generalized Linear Models were used for the examinations.

**Results:**

In addition to the apparent significant positive correlation of malaria cases with annual rainfall and mean temperature, several correlations were found for habitat parameters. The cover of marshlands in the 19th-century habitat structure of Central European quadrates was considerably the same as in the recent African ones. The extent of rural residential areas was significantly smaller in the 19th-century habitat structure of Central European quadrats than in present-day African ones. According to the revealed correlations, the surface cover of rural residential areas is the main driver of the number of autochthonous malaria cases that we can directly impact.

**Conclusions:**

The study confirmed with historical comparison that not only the annual rainfall and mean temperature, the cover of marshlands and other habitats with breeding sites, but also the elements of the rural human environment play a significant role in the high number of autochthonous malaria cases, probably through the concentration and enhancing sites for vector mosquitoes. The latter confirms that a rapid urbanization process could reduce malaria cases in the most infected areas of Africa. Until the latter happens, extensive biological control of *Anopheles* larvae and chemical control (both outdoor and indoor) of their imagoes, further mosquito nets, repellents, and carbon dioxide traps will need to be applied more widely in the most heavily infested areas.

**Supplementary Information:**

The online version contains supplementary material available at 10.1186/s12936-024-05004-y.

## Background

Malaria is still a worldwide health issue, mainly affecting countries in Africa and Asia, where the most susceptible age group is children under five years of age; almost 70% of the victims come from the above age group [1]. Eradication programmes significantly reduced the number of malaria cases worldwide between 1955 and 1972, but then new foci emerged at the turn of the millennium with annual deaths of around 1 million [2]. Following the improvement in recent decades (annual mortality has fallen from 1 million to 400–500 thousand worldwide), the emergence of COVID-19 has created a new situation. Because of the similarity of symptoms, malaria and COVID-19 infections were difficult to distinguish. Further, the restricting movements disturbed the success of malaria control programmes, resulting in significantly (+ 6–7%) more malaria cases and deaths [3–6].

There is cause for optimism that malaria has been totally eradicated in many parts of the world. Much of Europe used to be heavily infected with malaria historically, but today, it is considered an area free of endemic transmissions. In recent years, autochthonous transmissions have occurred only in parts of Eastern Europe belonging to Russia [7, 8]; elsewhere, only introduction cases have been known. Many factors, such as land use, housing quality, urbanization, and climate change, played a role in the overall curbing malaria in Western and Central Europe [9].

Spatial and temporal comparison of malaria hotspots and coldspots based on entomological, ecological, and epidemiological data [10] can improve the health outcomes of malaria control and reduction strategies [11].

Historically, Hungary, occurring mainly in a basin bottom, is probably the most water-rich region in Central Europe and has been consequently a malaria-endemic area for centuries. According to the map of Hollaender [12], about 120 years ago, in 26% of the country, more than 50 autochthonous malaria cases per 10,000 citizens per year were detected. Thanks to the launch of the eradication programme, malaria infection decreased significantly by the period of World War II. Still, as a result of the spreading effect of the war (general deterioration of hygienic conditions), it became significant again by 1948. At that time, 9% of Hungary was characterized by more than 50 cases/10,000 citizens/year [13]. Another rigorous eradication programme began in 1949. Treatment with DDT suspension was applied in all buildings (dwellings, stables, sheds) of all settlements, which were repeated for three consecutive years. The interventions (using the form of DDT and drainage of the water-covered habitats) were also extended to breeding sites of *Anopheles* mosquitoes. The intense interventions produced excellent results [14], as since 1956, only imported malaria cases have been present in the country (1–20 cases/year) [15]. Malaria morbidity in a given year was predicted by the degree of stagnant water cover of the previous year when considering the period before the launch of a drastic mosquito control programme by DDT, while such a relationship could not be revealed for a later period [16].

Unlike in Europe and North America, malaria is still a significant public health problem, for example, in sub-Saharan Africa [17]. The continuous breeding of *Anopheles* generations supports the spread of malaria in West, Central, and East Africa. The malaria hotspots are usually characterized by favourable conditions for the survival and activity of the *Anopheles* species including humidity of around 90% and a daily temperature range of 16 °C to 36 °C [18, 19]. Another main driver of the high density of malaria vectors is the surface cover of shallow temporary breeding sites [20]. So, precipitation is also considered a crucial climatic factor influencing malaria incidence [21, 22].

The level of malaria risk also depends on socio-ecological conditions. Increasing levels of urbanization, declining livestock numbers, modernization of animal husbandry, and separation of human and farm-animal habitats significantly reduce malaria transmission [23, 24]. According to the expectations, by 2040, over half of the African population will live in urban areas [25], which could solve the problem of malaria elimination in still severely affected sub-Saharan Africa.

To test the latter hypothesis, the relationship between malaria cases and background variables in 2 km × 2 km sized quadrates (10 Central European and 10 African) were examined. In addition to the annual precipitation, annual mean temperature, and the current habitat structure of the African sites, data on the above parameters detected in the nineteenth and twentieth centuries and currently in the Central European sites were included in the analyses (n = 40). The study sought to answer the following questions: (i) How has the suitability of habitat structure for the breeding of mosquitoes in the Central European quadrats changed over the last 120–130 years? (ii) Which background variables show a spatially and temporally independent correlation with the number of malaria cases?

## Methods

### Study sites

**Fig. 1:**
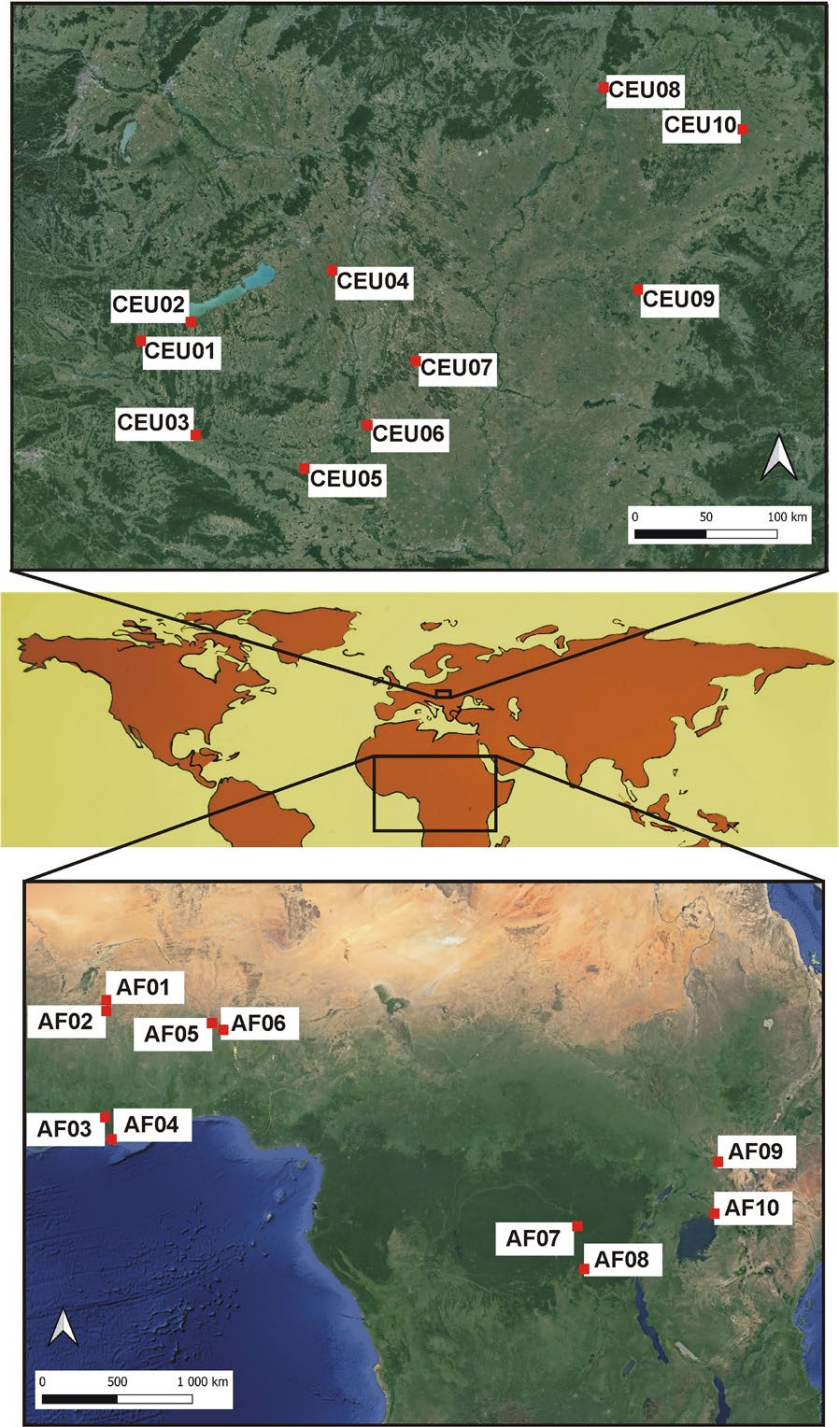
The study areas and the location of the 20 quadrates in Central Europe and Africa

To study the habitat structure and weather conditions of the Central European and African sites, ten quadrates in Central Europe (Hungary) and ten quadrates in Africa (Burkina Faso, Congo, Ghana, Ivory Coast, Niger, Nigeria, Uganda) were randomly selected (Fig. 1). To avoid autocorrelation, all the Central European sites were established in different mesoregions of the country ([26], see in Suppl. Materials, Figure S1). African sites were placed in seven countries. Each quadrate was 2 km × 2 km sized and covered an area of 400 ha (the coordinates of the quadrate centres are in Suppl. Materials, Table S1).

### Data collection

**Fig. 2:**
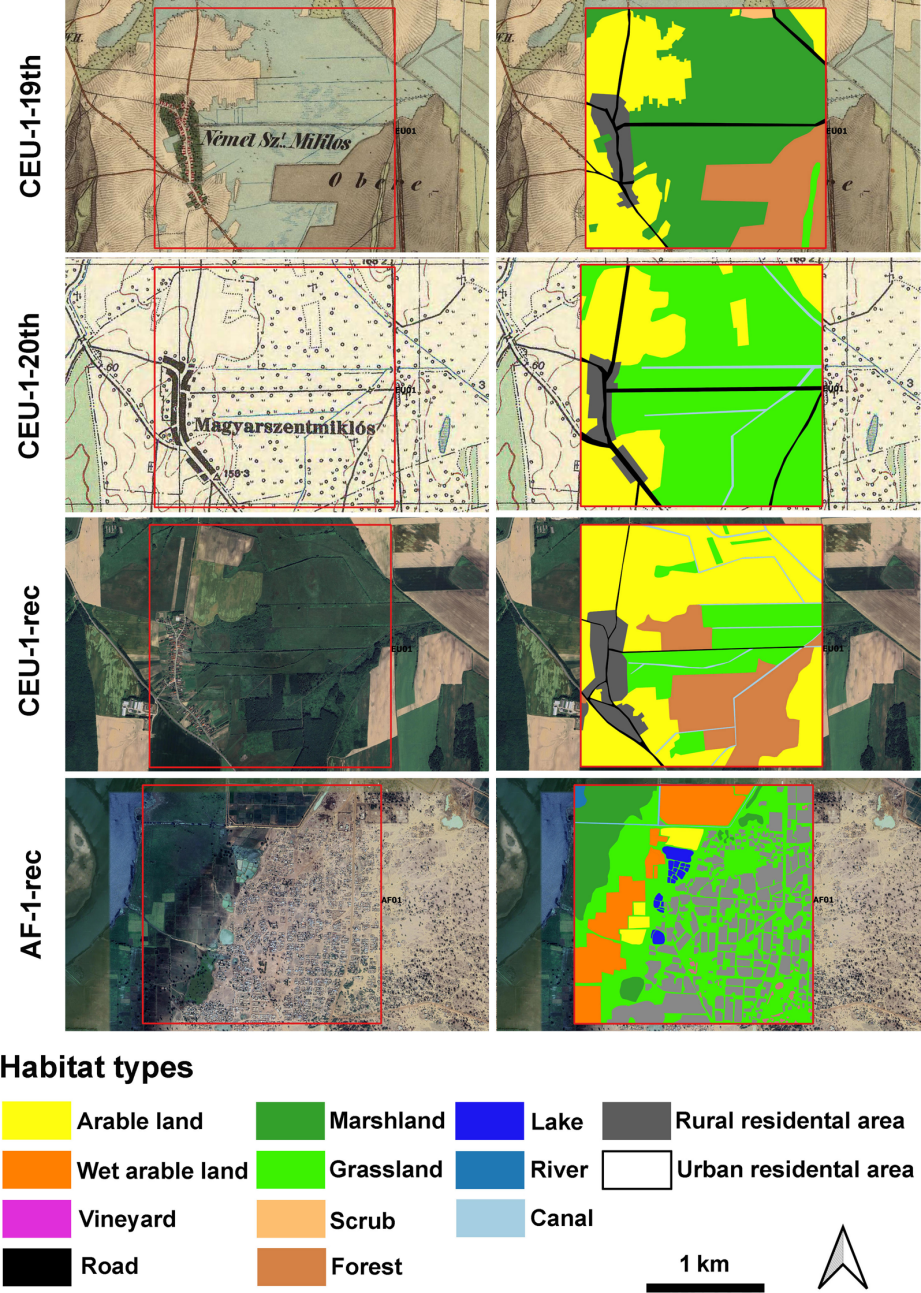
Examples for habitat maps of the study sites (CEU01 and AF01 sites, all maps see in Suppl. Mat. Figure S2a–S2m)

Surface cover per studied quadrates of the following habitat types was determined using QGIS 3.16.1 [27]: arable land, canal, forest, grassland, lake, marshland, river, road, rural residential area, scrub, urban residential area, vineyard (and wet arable land which restricted to the African sites) (Fig. 2).

The habitat maps of the Central European study sites for the following periods were drawn: (a) second half of the nineteenth century (CEU19th), (b) the mid of the twentieth century (CEU20th), (c) recent time (CEUrec). For the habitat mapping digital on-line maps of the Second military survey of the Habsburg Empire (Hungary, 1819–1869) [28] (https://mapire.eu), the Military survey of Hungary (1941) [29] (https://mapire.eu), and Google Earth (GE) CNS/Airbus files created on August 10, 2020; March 8, 2021; January 24, 2022; June 4, 2022; August 10, 2022; September 13, 2023; October 13, 2023; October 16, 2023; February 27, 2024; February 28, 2024 were used. Malaria cases per 10,000 citizens were added to the database based on works of Hollaender [12] (a: second half of the nineteenth century) and Mihályi and Gulyás [13] (b: mid of the twentieth century) (c: there is currently no autochthonous infection in the country).

The current habitat maps of the African sites (AFrec) are based on the following Google Earth (GE) CNS/Airbus files created on December 3, 2020; December 4, 2020; May 16, 2022; January 19, 2023; April 1, 2023; July 18, 2023; August 5, 2023; November 27, 2023; January 5, 2024; January 30, 2024. Malaria cases per 10,000 citizens were added to the African quadrates based on the Malaria Atlas Project (https://data.malariaatlas.org/).

In the African quadrates, annual precipitation using WorldClim (https://worldclim.org/) and mean temperature using Biological Forecasting and Hindcasting Tools [30] were determined. In the Central European quadrates, annual rainfall and mean temperature for the nineteenth and twentieth centuries with the use of databases of the Hungarian Meteorological Service (https://met.hu/) for the recent time (2023) with the use of databases of MetNet Hungary (https://www.metnet.hu/) were determined.

### Statistical analyses

Using QGIS, the surface cover (in square meters) and relative surface cover (cover of the habitat type per quadrate size) of the habitat types per site was calculated. The fundamental differences in quadrate landscape structure were determined by box-plot diagrams of the relative surface cover of the habitat types on the maps of CEU19th, CEU20th, CEUrec, and AFrec. Mann–Whitney tests were used to test the relationships between the data series.

Differences between the habitat structure of each quadrat (n = 40) were also examined using PCA analysis (relative surface cover values, correlation matrix, and biplot interpretation).

After normality tests, generalized linear models (GLM) by assuming a normal distribution to assess the association between background variables and malaria cases were built. To meet the statistical assumptions, the number of malaria cases, values of annual precipitation and mean temperature were log-transformed. Relative surface cover values of the habitat types were left untransformed. Cases where any of the variables showed zero values were omitted. Statistical procedures were performed by using the PAST 2.16 [31] software package.

## Results

**Fig. 3:**
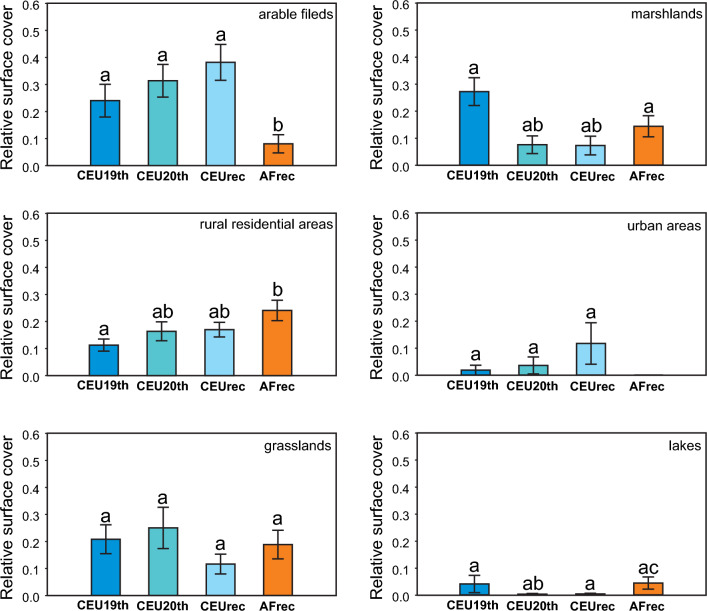
Differences of relative surface cover of the most characteristic habitat types in the studied quadrates: mean values (± SE) in CEU19th (n = 10), CEU20th (n = 10), CEUrec (n = 10), and AFrec (n = 10) sites. Statistically significant (p < 0.05) differences detected using Mann–Whitney tests are indicated by different letters

Based on the pooled data of the habitat types (Fig. 3) that were characteristic in the study areas, grassland and lake surface cover showed no significant differences in either space (CEU vs. AF) or time (CEU19th vs. CEU 20th vs. CEUrec). Urban areas were present in only a tiny area of the African quadrats (AFrec). The habitat type showed a non-significant increase in European quadrates from the nineteenth century to now. The extent of rural residential areas was significantly smaller in the 19th-century habitat structure of Central European quadrats (CEU19th) than in present-day African ones (AFrec). The extent of marshlands in the 19th-century habitat structure of Central European quadrates (CEU19th) was significantly the same as the recent African ones (AFrec). In the habitat structure of the Central European sites recorded in the twentieth century and recent times, the surface cover of the marshlands was significantly lower than that of CEU19th and AFrec. The extent of arable land in the African study areas (AFrec) is significantly smaller than that of any period in Central Europe (CEU19th, CEU 20th, CEUrec).

**Fig. 4:**
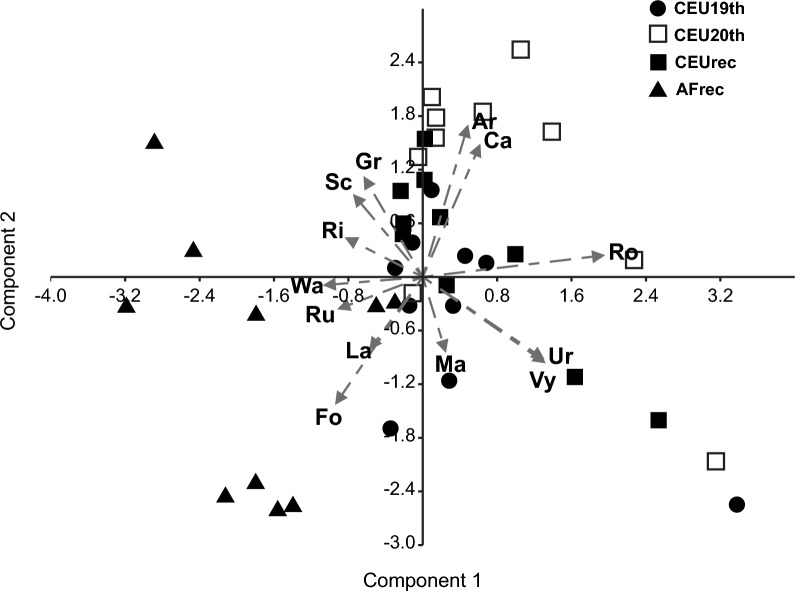
PCA analysis detected differences in the habitat structure of the studied quadrats. African study sites (AF) showed isolation mainly in the presence of forest (Fo), lake (La), wet arable land (Wa), and rural residential area (Ru) habitat types. CEU quadrats from the nineteenth century differed from other sites in the high relative surface cover of arable fields (Ar) and canals (Ca) (n = 40). Abbreviations of the habitat types: Ar: arable land, Ca: canal, Fo: forest, Gr: grassland, La: lake, Ma: marshland, Ri: river, Ro: road, Ru: rural residential area, Sc: scrub, Ur: urban residential area, Vy: vineyard, Wa: wet arable land

Results of the PCA analysis (Fig. 4) (based on relative surface cover values of the detected habitat types, distribution of the eigenvalues was the following: > 1: 6, 0.75–1: 2, 0,50–0.75: 2, 0.30–0.50: 2, < 0.30: 1) showed the isolation of the majority of the African study sites (AF), with only two sites (AF02, AF06) being classified into the group of the CEU19th sites. The distinctiveness of the AF sites is mainly due to the strong presence of the forest (Fo), lake (La), wet arable land (Wa), and rural residential area (Ru) habitat types. The CEU quadrats in the PCA figure showed isolation mainly in the 20th-century quadrats, with arable fields (Ar) and canals (Ca) showing a prominent presence. The habitat structure of CEU03rec and CEU07 of all periods (19th, 20th, rec) still showed a separation. In the latter case, the significant surface cover of vineyards (Vy) and urbanised areas (Ur) provided the basis for similarity.

**Fig. 5:**
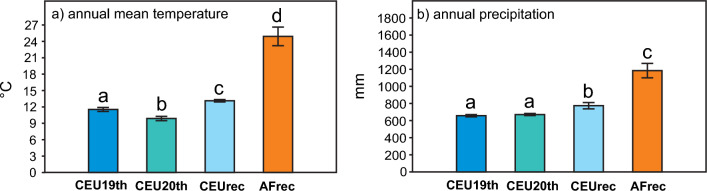
Differences of annual mean temperature (**a**) and annual precipitation (**b**) in the studied quadrates: mean values (± SE) in CEU19th (n = 10), CEU20th (n = 10), CEUrec (n = 10), and AFrec (n = 10) sites. Statistically significant (p < 0.05) differences detected using Mann–Whitney tests are indicated by different letters

Not only the mean annual temperature of the Central European (CEU) quadrats differed significantly from the Africans (AFrec), but the mean annual temperature values for the Central European quadrats at different times (CEU19th, CEU 20th, CEUrec) too (Fig. 5.). The yearly precipitation was significantly higher in the African quadrates (AFrec). Further, in the Central European quadrates, the annual rainfall was significantly higher in the recent time (CEUrec), without autochthonous malaria cases, than in former times (CEU19th, CEU 20th), before the eradication (Fig. 5).

**Fig. 6:**
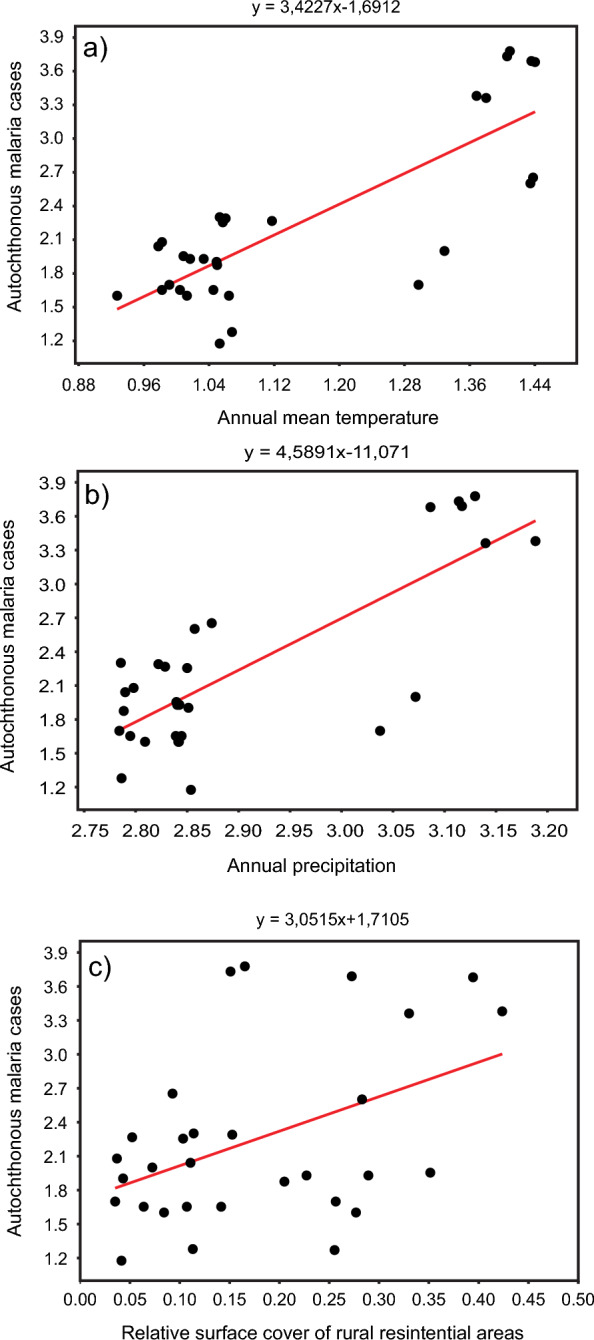
GLM analyses detected robust significant correlations between **a** the annual mean temperature, **b** the annual precipitation, **c** the surface cover of rural residential areas (Ru) and the number of autochthonous malaria cases (n = 30) (all data were log-transformed)

GLM analyses searching for correlations between the background variables and malaria cases showed that over the annual precipitation and mean temperature, the surface cover of rural residential areas (Ru) drove the number of autochthonous malaria cases (Fig. 6).

## Discussion

The results of the present study on determining malaria cases by background variables provide a reasonable basis for a perspective assessment despite the significant climatic differences between the Central European and African study quadrats. Share of the marshland habitat type, significant for breeding malaria vector mosquitoes, showed a high degree of similarity between the habitat structure of the Central European quadrats in the nineteenth century and currently in the African studied ones. In contrast to African areas, where climatic conditions ensure the maintenance of all-year continuous breeding of malaria-transmitting mosquito species, the decline of the marshland habitat type in Central Europe in the twentieth century should be closely linked to the deterioration of autochthonous malaria cases. Another important finding of the study is that temporal (CEU) and spatial (CEU vs. AF) analyses of potential drivers of malaria cases showed that not only the annual temperature and precipitation but also the surface cover of rural residential areas (Ru) plays an essential role in the number of autochthonous malaria cases.

Based on the above results, there are severe landscape structural reasons why more than pesticide mosquito control is required. The situation is exacerbated when a country has no resources for chemical control either [32]. The land use, including drainage, involvement in cultivation, and diminishing irrigated crops in the potential breeding sites of malaria vectors, strongly supported successful local eradication [33, 34]. Swarming of mosquitoes breeding in marshlands, such as *Anopheles* species, cover an area of a 2000 m radius [35]. Hence, the above interventions are sufficient to protect much larger areas than they take. In Hungary, the development of the sewerage network for the drainage of inland waters significantly decreased the periodically water-covered areas. In 1935, the early infrastructure with a total length of 14,300 km [36] significantly reduced the extent of breeding sites formed after heavy rains and floods, but later, its coverage increased sharply. Today, the total length of the network of canals reaches 42,400 km (QGIS calculation after data from https://map.mbfsz.gov.hu/).

Wet arable lands, which are well suitable for mosquito breeding habitats [37] and characteristic in some African study quadrates, have also been present in the Central European study area. Around 1900, rice was cultivated on 1200 hectares in Hungary, but this figure fell utterly, and in 1939 rice cultivations covered only 28 hectares (from the 98,000 square km of the country) [38]. During the Second World War, the area under rice production increased significantly (in 1944, it was 5000 hectares), and then the increase jumped in the 1950s (in 1953, it was 28,000 hectares) [39]. The latter is also the most intensive period of the malaria eradication programme. From 1956 onwards, only introduced malaria cases occurred, even though the area under rice was 50,000 hectares until 1970, when it gradually declined to the current 2000–3000 hectares [40].

*Anopheles* species, the primary vector of malaria, in Central Europe do not breed continuously; there is a winter break in the line of the generations. In Hungary, *Anopheles* mosquitoes overwinter mainly in stables, and the wintering individuals play a prominent role in transmitting the disease [16, 41]. It is not only the drastic use of pesticides that may have played a role in eradicating the local autochthonous malaria cases. In the pre-eradication period (1935), livestock farming (and, accordingly, the number of stables) was exceptionally high in the southwestern areas of Hungary, one of the regions affected mainly through malaria [42]. From the period above to the present day, the horse population in the country has decreased drastically (from ~ 900,000 to less than 100,000), and the cattle population has more than halved (from > 2 million to 1 million >) [43]. The overall effect of the above changes in cattle and horse stables has led to a drastic reduction in the number of buildings suitable for the mass overwintering of the females of *Anopheles* mosquitoes.

The level of precipitation, like in this study, has usually been considered the most important climatic factor influencing autochthonous malaria cases [21, 22]. *Anopheles* mosquitoes lay their eggs on the water surface, which is ideally suited to both permanent water bodies and fresh rainwater puddles. The ecology of former and potential vector species of malaria, which are well distributed even after the eradication, in the Central European quadrates is well known [44]. *Anopheles maculipennis *sensu lato (*s.l*.) is common in the hilly and mountainous areas of Hungary, is rare in the plains, and usually absent in the saline areas. Its females are zoophilic (feed mainly on cattle, secondarily on pig and poultry) and very slightly anthropophilic. *Anopheles messeae* is widespread in CEU sites, mainly in lowland areas, its females are zoophilic (feed mainly on mammalian) and anthropophilic. Occurrence of *Anopheles atroparvus* in Central European sites is also typical in the lowlands; females are zoophilic (feed mainly on mammalians) and anthropophilic. *Anopheles plumbeus* breeds specifically in tree holes, the species is widespread in forest areas. Its females are highly anthropophilic but also zoophilic (mammals, birds, reptiles). In the Central European quadrates, *Anopheles hyrcanus,* considered the most potential malaria vector, is common in Hungary and mainly found around large lakes. Females of *An. hyrcanus* are also highly anthropophilic, while it is also zoophilic (mammals).

The result of this study, that over the annual temperature and precipitation, the surface cover of rural residential areas drives the number of autochthonous malaria cases, fits in well with previous statements that number of malaria cases is generally lower in urban than rural areas [23, 24, 45–47]. In contrast, the recent examinations did not confirm the statement [48, 49] that the rural human environment is richer in breeding sites for vector *Anopheles* species than others. In the studied sites, surface cover of marshland (Ma), wet arable land (Wa), and lake (La) habitats, having very suitable breeding sites for *Anopheles* larvae, did not show significant correlations with surface cover of rural residential areas (Ru). Still, malaria cases are based on the presence and extension of *Anopheles* breeding sites, but the proportion of the rural residential area (Ru) in their upsurge is impressive. Farm buildings, stables, and livestock belonging to the rural environment seem to be important concentration and enhancing sites for vector mosquitoes, where they can diverge to alternative hosts such as bovines, sheep, and goats [50].

The findings of this study provided additional information that could help reduce malaria cases in Africa, similar to former eradications in Europe. In the topic, the following activities have particular importance: (a) intensive and systematic biological control (proposed technology: aerial granulation) in extensive breeding sites of *Anopheles* species occurring within 2 km of the populated areas, (b) regular chemical control of *Anopheles* mosquitoes outdoor and indoor in livestock buildings with insecticides, (c) using mosquito nets at all buildings as dispersed as possible. Based on historical examples of Central Europe, the above-mentioned interventions could effectively reduce malaria cases in most endemic areas of Africa. Still, the recent shift in transmission from indoors to outdoors [50–52] makes it difficult to achieve quick results. The latter makes (d) the use of repellents to protect the population, possibly including high-efficiency outdoor CO_2_ traps, essential to achieve a meaningful reduction in malaria cases.

## Conclusions

By comparing historical data from Central Europe with current data collected in Africa, the present study confirmed that the extensive rural human environment plays a significant role in the high number of autochthonous malaria cases. The latter confirms the scenario suggested by several studies that a rapid urbanization process could reduce malaria cases in the most affected areas of Africa. However, in the current economic situation, neither extensive urbanization nor rapid and general livestock farming modernization seems promising in Africa. Therefore, to reduce morbidity and mortality even more, the following implications should be widely implemented, with essential international support, in the most malaria-affected areas of Africa: extensive aerial biological control of *Anopheles* species breeding sites within 2 km of the populated regions, regular chemical control of *Anopheles* imagoes both outdoor and indoor, using mosquito nets at all buildings, repellents and high-efficiency outdoor CO_2_ traps to protect the population from the outdoor infections.

### Supplementary Information

Supplementary Material 1: **Table S1**. Coordinates of the centre of the studied quadrates. **Table S2a-j**. Relative surface cover of the recorded habitat types (abbreviations see in the manuscript), Malaria cases (MC) per 10,000 citizens, annual mean temperature (MT) and annual precipitation (AP) in CEU01–CEU10 study sites. **Table S2k**. Relative surface cover of the recorded habitat types (abbreviations see in the manuscript) in AF01, AF02, AF03, AF04 study sites. **Table S2l**. Relative surface cover of the recorded habitat types (abbreviations see in the manuscript) in AF05, AF06, AF08, AF08 study sites. **Table S2m**. Relative surface cover of the recorded habitat types (abbreviations see in the manuscript) in AF09, AF10 study sites. **Table S2n**. Malaria cases (MC) per 10,000 citizens, annual mean temperature (MT) and annual precipitation (AP) in the African study sites. **Figure S1**. Central European quadrate in different mesoregions of the country. **Figure S2a-j**. Habitat maps of the CEU01–CEU10 study sites. **Figure S2k**. Habitat maps of the AF01, AF02, AF03, AF04 study sites. **Figure S2l**. Habitat maps of the AF05, AF06, AF07, AF08 study sites. **Figure S2m**. Habitat maps of the AF09, AF10 study sites.

## Data Availability

The original data are available in the online version.
