# Supplementary material for: Drivers of autochthonous malaria cases over time: could the Central European present the African future?
Source: Malar J. 2024 Jun 10;23:181. doi: 10.1186/s12936-024-05004-y (PMC11163750; doi:10.1186/s12936-024-05004-y)
Supplement: Supplementary file 1 — Supplementary Material 1: Table S1. Coordinates of the centre of the studied quadrates. Table S2a-j. Relative surface cover of the recorded habitat types (abbreviations see in the manuscript), Malaria cases (MC) per 10,000 citizens, annual mean temperature (MT) and annual precipitation (AP) in CEU01–CEU10 study sites. Table S2k. Relative surface cover of the recorded habitat types (abbreviations see in the manuscript) in AF01, AF02, AF03, AF04 study sites. Table S2l. Relative surface cover of the recorded habitat types (abbreviations see in the manuscript) in AF05, AF06, AF08, AF08 study sites. Table S2m. Relative surface cover of the recorded habitat types (abbreviations see in the manuscript) in AF09, AF10 study sites. Table S2n. Malaria cases (MC) per 10,000 citizens, annual mean temperature (MT) and annual precipitation (AP) in the African study sites. Figure S1. Central European quadrate in different mesoregions of the country. Figure S2a-j. Habitat maps of the CEU01–CEU10 study sites. Figure S2k. Habitat maps of the AF01, AF02, AF03, AF04 study sites. Figure S2l. Habitat maps of the AF05, AF06, AF07, AF08 study sites. Figure S2m. Habitat maps of the AF09, AF10 study sites. [file 12936_2024_5004_MOESM1_ESM.docx]

**Supplementary Material for**

Drivers of autochthonous malaria cases over time – Could the Central European present the African future?

Zoltán Kenyeres

**Table S1**. Coordinates of the centre of the studied quadrates.

| **Quadrate** | **Country** | **Coordinates** |
| --- | --- | --- |
| AF01 | Burkina Faso | N13°10'31.99"; W3°24'40.39" |
| AF02 | Burkina Faso | N12°58'42.49"; W3°26'30.93" |
| AF03 | Ivory Coast | N6°21'46.61"; W3°24'46.87" |
| AF04 | Ghana | N 5°4'56.07"; W2°56'12.87" |
| AF05 | Niger | N12° 4'11.41"; E3°10'55.65" |
| AF06 | Nigeria | N11°38'36.16"; E3°36'57.10" |
| AF07 | Congo | S0°21'24.03"; E25°25'59.90" |
| AF08 | Congo | S2°54'48.90"; E25°52'56.10" |
| AF09 | Uganda | N3°31'41.23"; E34° 7'32.12" |
| AF10 | Uganda | N0°27'45.70"; E33°54'17.36" |
| CEU01 | Hungary | N46°32'26.13"; E16°56'45.26" |
| CEU02 | Hungary | N46°40'30.02"; E17°23'39.46" |
| CEU03 | Hungary | N45°57'33.29"; E17°27'34.85" |
| CEU04 | Hungary | N46°59'51.63"; E18°42'16.22" |
| CEU05 | Hungary | N45°44'54.49"; E18°27'48.27" |
| CEU06 | Hungary | N46° 1'45.24"; E19° 2'11.23" |
| CEU07 | Hungary | N46°25'44.28"; E19°28'19.16" |
| CEU08 | Hungary | N48° 8'34.16"; E21°14'48.07" |
| CEU09 | Hungary | N46°51'56.35"; E21°31'33.00" |
| CEU10 | Hungary | N47°51'4.41"; E22°32'44.94" |

**Figure S1**. Central European quadrate in different mesoregions of the country.


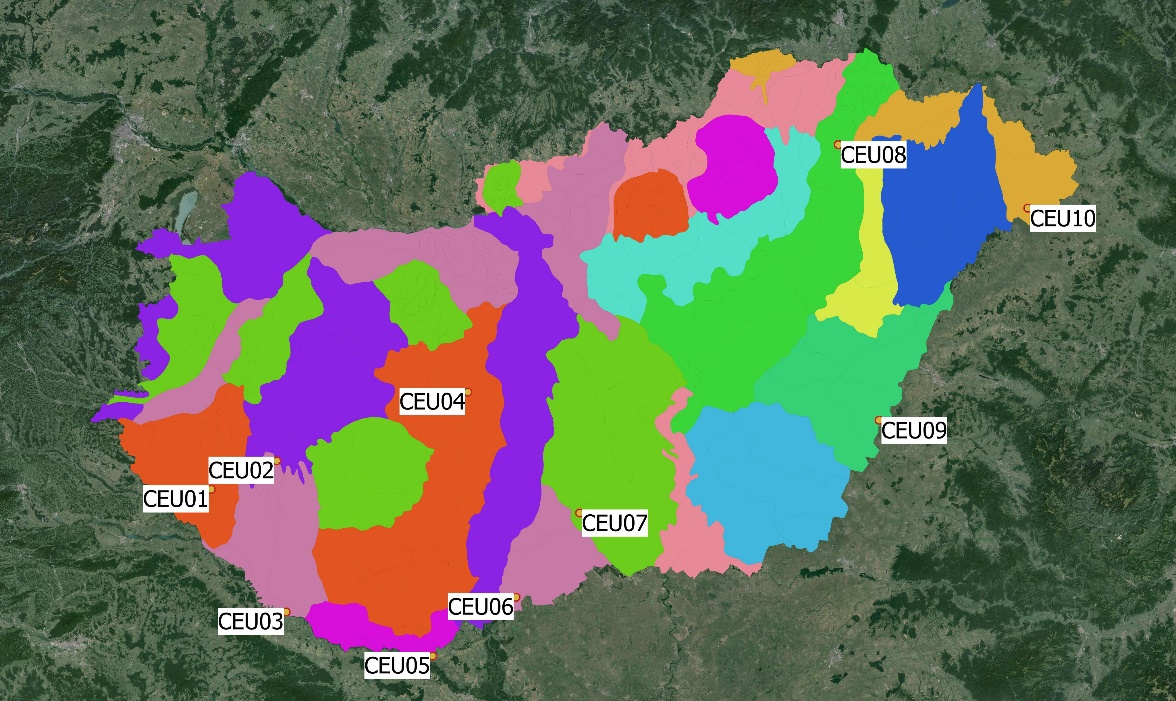


**Figure** **S2a**. Habitat maps of the CEU01 study site.


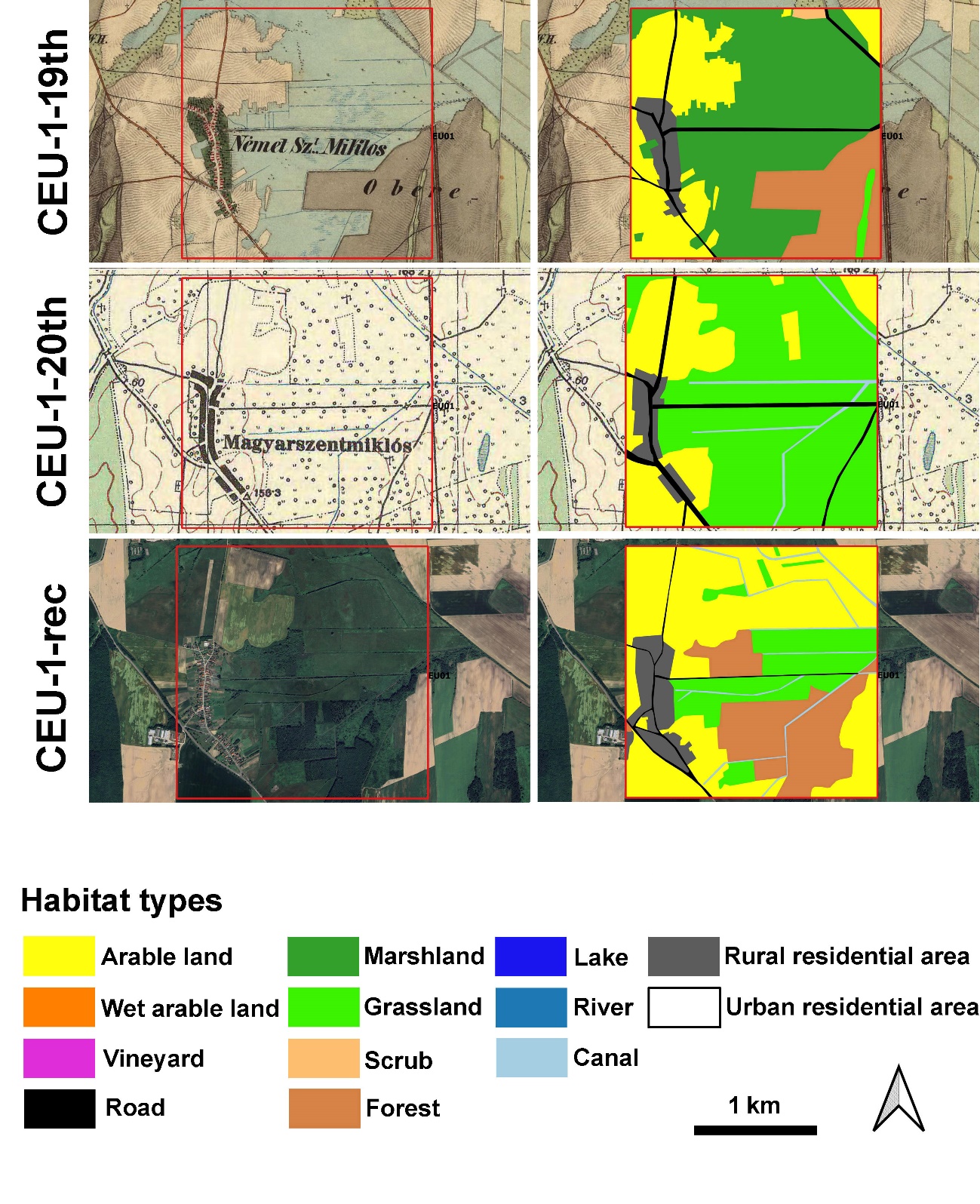


**Table S2a**. Relative surface cover of the recorded habitat types (abbreviations see in the manuscript), Malaria cases (MC) per 10,000 citizens, annual mean temperature (MT) and annual precipitation (AP) in CEU01 study site.

|  | **Ar** | **Ca** | **Fo** | **Gr** | **La** | **Ma** | **Ri** | **Ro** | **Ru** | **Sh** | **Ur** | **Wa** | **Wy** |
| --- | --- | --- | --- | --- | --- | --- | --- | --- | --- | --- | --- | --- | --- |
| **19^th^** | 0.246 | 0.000 | 0.153 | 0.010 | 0.000 | 0.513 | 0.000 | 0.031 | 0.041 | 0.005 | 0.000 | 0.000 | 0.000 |
| **20^th^** | 0.239 | 0.022 | 0.000 | 0.657 | 0.000 | 0.000 | 0.000 | 0.045 | 0.037 | 0.000 | 0.000 | 0.000 | 0.000 |
| **rec** | 0.513 | 0.026 | 0.206 | 0.172 | 0.000 | 0.000 | 0.000 | 0.016 | 0.068 | 0.000 | 0.000 | 0.000 | 0.000 |

|  | **MC** | **MT (°C)** | **AP (mm)** |
| --- | --- | --- | --- |
| **19^th^** | 15 | 11.3 | 714.0 |
| **20^th^** | 120 | 9.6 | 628.1 |
| **rec** | 0 | 12.6 | 894.6 |

**Figure S2b**. Habitat maps of the CEU02 study site.


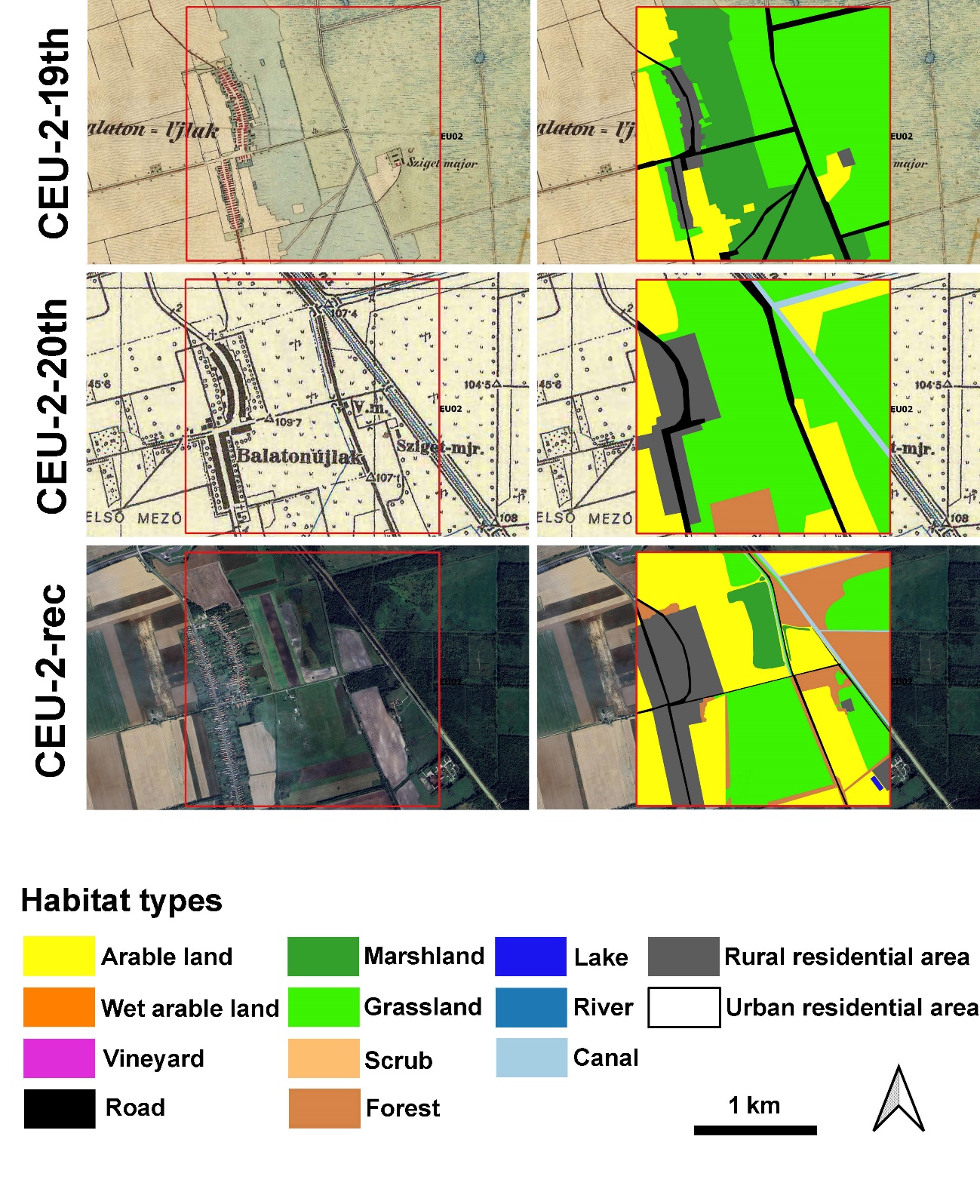


**Table S2b**. Relative surface cover of the recorded habitat types (abbreviations see in the manuscript), Malaria cases (MC) per 10,000 citizens, annual mean temperature (MT) and annual precipitation (AP) in CEU02 study site.

|  | **Ar** | **Ca** | **Fo** | **Gr** | **La** | **Ma** | **Ri** | **Ro** | **Ru** | **Sh** | **Ur** | **Wa** | **Wy** |
| --- | --- | --- | --- | --- | --- | --- | --- | --- | --- | --- | --- | --- | --- |
| **19^th^** | 0.170 | 0.000 | 0.000 | 0.454 | 0.000 | 0.266 | 0.000 | 0.066 | 0.043 | 0.000 | 0.000 | 0.000 | 0.000 |
| **20^th^** | 0.194 | 0.028 | 0.037 | 0.578 | 0.000 | 0.000 | 0.000 | 0.053 | 0.111 | 0.000 | 0.000 | 0.000 | 0.000 |
| **rec** | 0.352 | 0.017 | 0.157 | 0.255 | 0.001 | 0.042 | 0.000 | 0.027 | 0.150 | 0.000 | 0.000 | 0.000 | 0.000 |

|  | **MC** | **MT (°C)** | **AP (mm)** |
| --- | --- | --- | --- |
| **19^th^** | 80 | 11.2 | 710.0 |
| **20^th^** | 110 | 9.5 | 616.4 |
| **rec** | 0 | 13.7 | 816.2 |

**Figure S2c**. Habitat maps of the CEU03 study site.


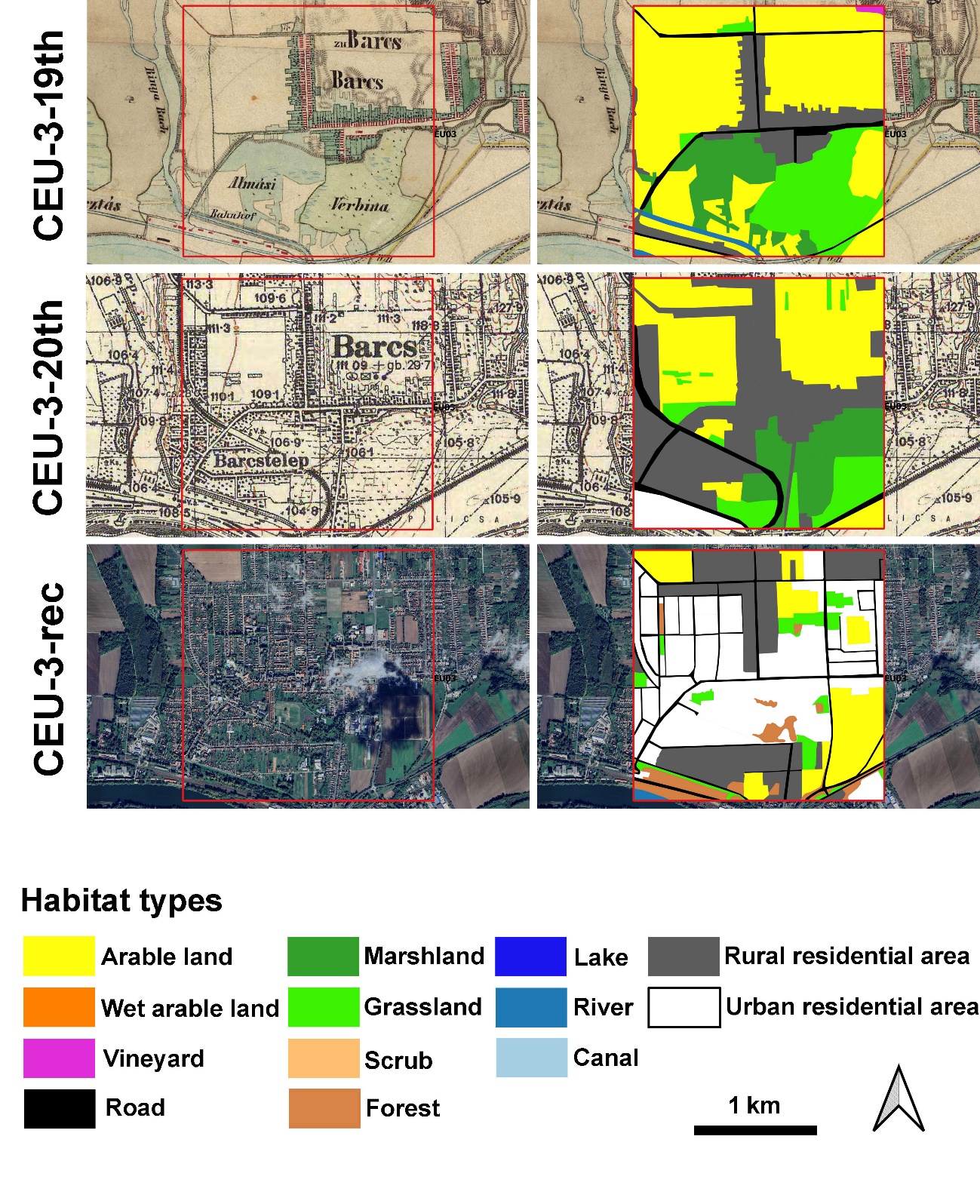


**Table S2c**. Relative surface cover of the recorded habitat types (abbreviations see in the manuscript), Malaria cases (MC) per 10,000 citizens, annual mean temperature (MT) and annual precipitation (AP) in CEU03 study site.

|  | **Ar** | **Ca** | **Fo** | **Gr** | **La** | **Ma** | **Ri** | **Ro** | **Ru** | **Sh** | **Ur** | **Wa** | **Wy** |
| --- | --- | --- | --- | --- | --- | --- | --- | --- | --- | --- | --- | --- | --- |
| **19^th^** | 0.575 | 0.000 | 0.000 | 0.151 | 0.000 | 0.102 | 0.012 | 0.044 | 0.114 | 0.000 | 0.000 | 0.000 | 0.003 |
| **20^th^** | 0.345 | 0.000 | 0.000 | 0.093 | 0.000 | 0.128 | 0.000 | 0.043 | 0.351 | 0.000 | 0.040 | 0.000 | 0.000 |
| **rec** | 0.151 | 0.000 | 0.050 | 0.043 | 0.000 | 0.000 | 0.004 | 0.089 | 0.212 | 0.000 | 0.450 | 0.000 | 0.000 |

|  | **MC** | **MT (°C)** | **AP (mm)** |
| --- | --- | --- | --- |
| **19^th^** | 200 | 11.3 | 610.4 |
| **20^th^** | 90 | 10.2 | 691.2 |
| **rec** | 0 | 13.1 | 930.5 |

**Figure S2d**. Habitat maps of the CEU04 study site.


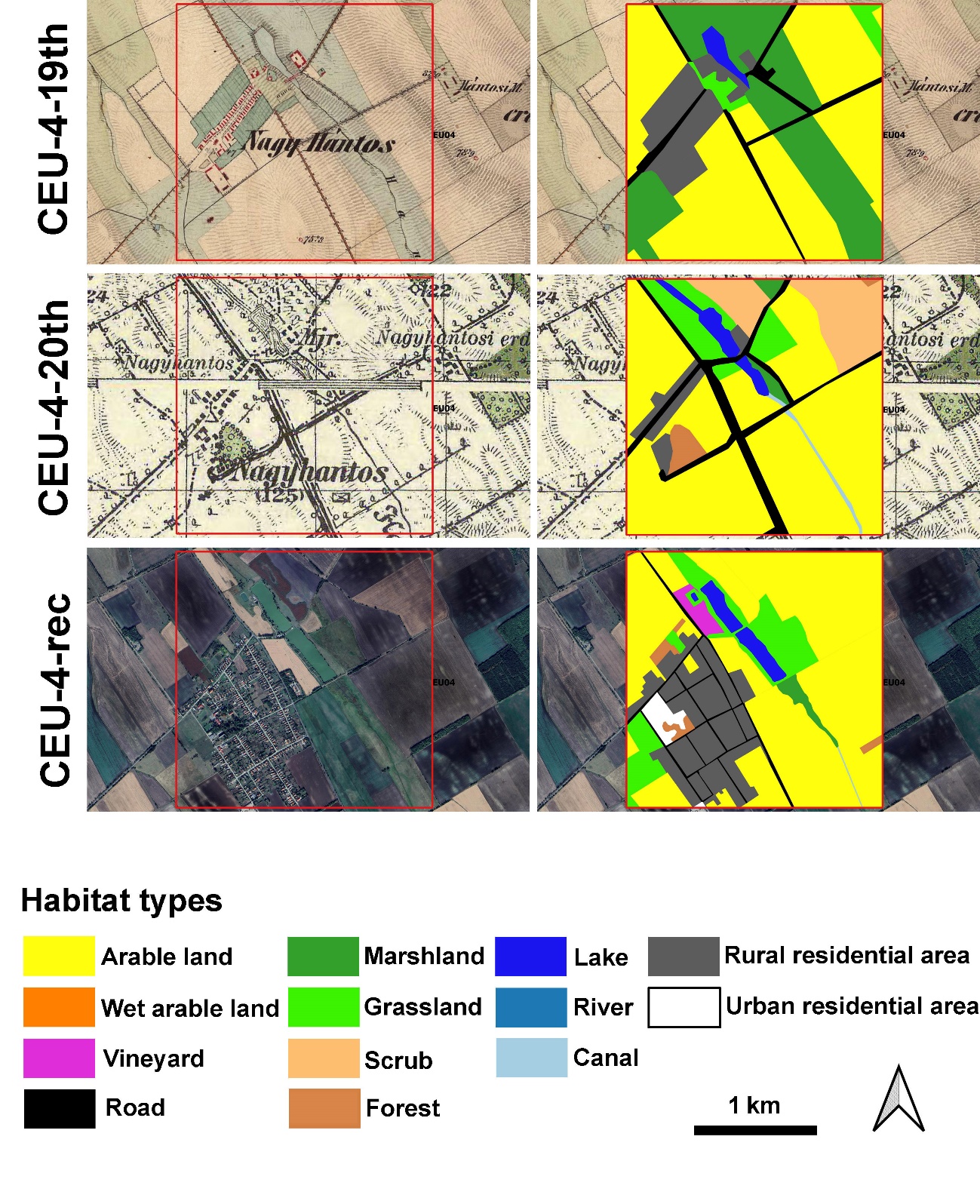


**Table S2d**. Relative surface cover of the recorded habitat types (abbreviations see in the manuscript), Malaria cases (MC) per 10,000 citizens, annual mean temperature (MT) and annual precipitation (AP) in CEU04 study site.

|  | **Ar** | **Ca** | **Fo** | **Gr** | **La** | **Ma** | **Ri** | **Ro** | **Ru** | **Sh** | **Ur** | **Wa** | **Wy** |
| --- | --- | --- | --- | --- | --- | --- | --- | --- | --- | --- | --- | --- | --- |
| **19^th^** | 0.529 | 0.000 | 0.000 | 0.026 | 0.013 | 0.281 | 0.000 | 0.048 | 0.103 | 0.000 | 0.000 | 0.000 | 0.000 |
| **20^th^** | 0.632 | 0.008 | 0.018 | 0.070 | 0.026 | 0.012 | 0.000 | 0.078 | 0.035 | 0.120 | 0.000 | 0.000 | 0.000 |
| **rec** | 0.612 | 0.001 | 0.014 | 0.106 | 0.027 | 0.012 | 0.000 | 0.029 | 0.169 | 0.000 | 0.017 | 0.000 | 0.014 |

|  | **MC** | **MT (°C)** | **AP (mm)** |
| --- | --- | --- | --- |
| **19^th^** | 180 | 11.4 | 708.0 |
| **20^th^** | 50 | 9.8 | 608.4 |
| **rec** | 0 | 13.0 | 703.4 |

**Figure S2e**. Habitat maps of the CEU05 study site.


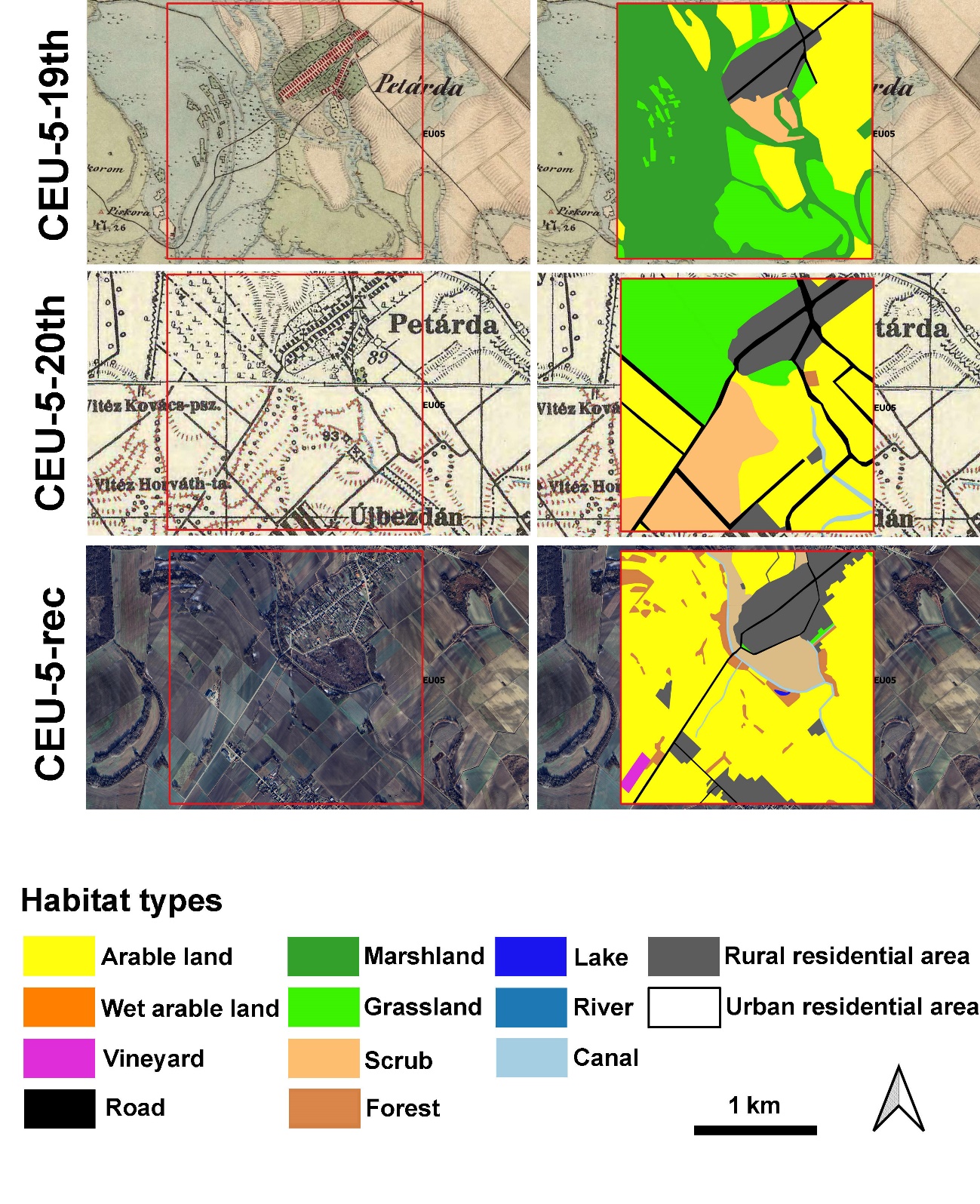


**Table S2e**. Relative surface cover of the recorded habitat types (abbreviations see in the manuscript), Malaria cases (MC) per 10,000 citizens, annual mean temperature (MT) and annual precipitation (AP) in CEU05 study site.

|  | **Ar** | **Ca** | **Fo** | **Gr** | **La** | **Ma** | **Ri** | **Ro** | **Ru** | **Sh** | **Ur** | **Wa** | **Wy** |
| --- | --- | --- | --- | --- | --- | --- | --- | --- | --- | --- | --- | --- | --- |
| **19^th^** | 0.246 | 0.000 | 0.000 | 0.189 | 0.000 | 0.463 | 0.000 | 0.010 | 0.064 | 0.029 | 0.000 | 0.000 | 0.000 |
| **20^th^** | 0.410 | 0.012 | 0.003 | 0.240 | 0.000 | 0.000 | 0.000 | 0.075 | 0.107 | 0.154 | 0.000 | 0.000 | 0.000 |
| **rec** | 0.705 | 0.014 | 0.048 | 0.002 | 0.001 | 0.000 | 0.000 | 0.017 | 0.143 | 0.063 | 0.000 | 0.000 | 0.008 |

|  | **MC** | **MT (°C)** | **AP (mm)** |
| --- | --- | --- | --- |
| **19^th^** | 45 | 11.1 | 623.4 |
| **20^th^** | 50 | 10.1 | 690.3 |
| **rec** | 0 | 13.2 | 946.9 |

**Figure S2f**. Habitat maps of the CEU06 study site.


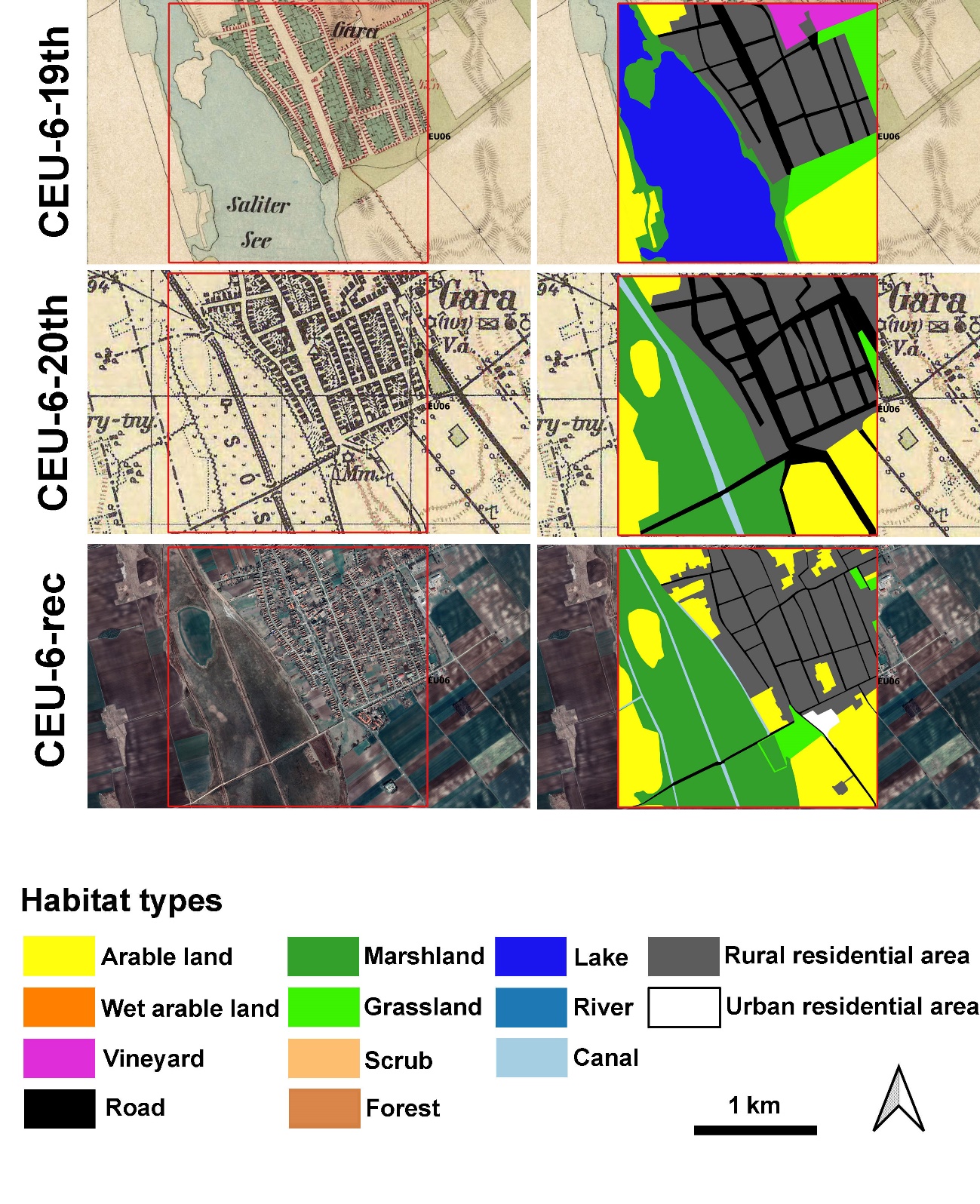


**Table S2f**. Relative surface cover of the recorded habitat types (abbreviations see in the manuscript), Malaria cases (MC) per 10,000 citizens, annual mean temperature (MT) and annual precipitation (AP) in CEU06 study site.

|  | **Ar** | **Ca** | **Fo** | **Gr** | **La** | **Ma** | **Ri** | **Ro** | **Ru** | **Sh** | **Ur** | **Wa** | **Wy** |
| --- | --- | --- | --- | --- | --- | --- | --- | --- | --- | --- | --- | --- | --- |
| **19^th^** | 0.158 | 0.000 | 0.000 | 0.081 | 0.324 | 0.079 | 0.000 | 0.068 | 0.255 | 0.000 | 0.000 | 0.000 | 0.035 |
| **20^th^** | 0.195 | 0.027 | 0.000 | 0.005 | 0.000 | 0.307 | 0.000 | 0.177 | 0.289 | 0.000 | 0.000 | 0.000 | 0.000 |
| **rec** | 0.252 | 0.022 | 0.000 | 0.024 | 0.000 | 0.326 | 0.000 | 0.044 | 0.323 | 0.000 | 0.008 | 0.000 | 0.000 |

|  | **MC** | **MT (°C)** | **AP (mm)** |
| --- | --- | --- | --- |
| **19^th^** | 40 | 11.6 | 644.4 |
| **20^th^** | 85 | 10.8 | 692.5 |
| **rec** | 0 | 13.2 | 784.8 |

**Figure S2g**. Habitat maps of the CEU07 study site.


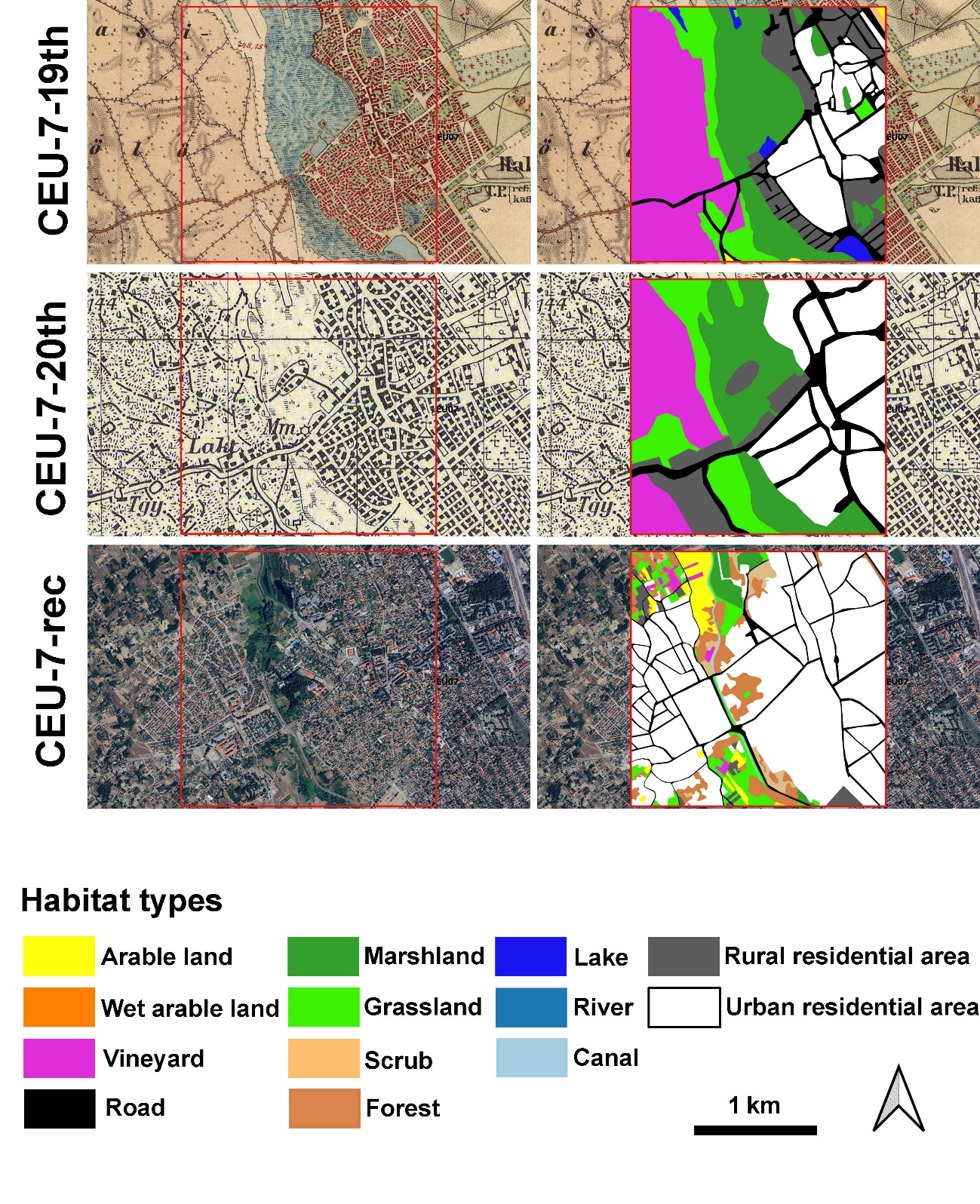


**Table S2g**. Relative surface cover of the recorded habitat types (abbreviations see in the manuscript), Malaria cases (MC) per 10,000 citizens, annual mean temperature (MT) and annual precipitation (AP) in CEU07 study site.

|  | **Ar** | **Ca** | **Fo** | **Gr** | **La** | **Ma** | **Ri** | **Ro** | **Ru** | **Sh** | **Ur** | **Wa** | **Wy** |
| --- | --- | --- | --- | --- | --- | --- | --- | --- | --- | --- | --- | --- | --- |
| **19^th^** | 0.003 | 0.000 | 0.000 | 0.081 | 0.017 | 0.217 | 0.000 | 0.099 | 0.113 | 0.002 | 0.181 | 0.000 | 0.287 |
| **20^th^** | 0.000 | 0.000 | 0.000 | 0.123 | 0.000 | 0.197 | 0.000 | 0.106 | 0.084 | 0.000 | 0.314 | 0.000 | 0.176 |
| **rec** | 0.037 | 0.005 | 0.080 | 0.051 | 0.000 | 0.022 | 0.000 | 0.076 | 0.024 | 0.021 | 0.676 | 0.000 | 0.009 |

|  | **MC** | **MT (°C)** | **AP (mm)** |
| --- | --- | --- | --- |
| **19^th^** | 19 | 11.7 | 611.4 |
| **20^th^** | 40 | 10.3 | 695.1 |
| **rec** | 0 | 13.7 | 652.5 |

**Figure S2h**. Habitat maps of the CEU08 study site.


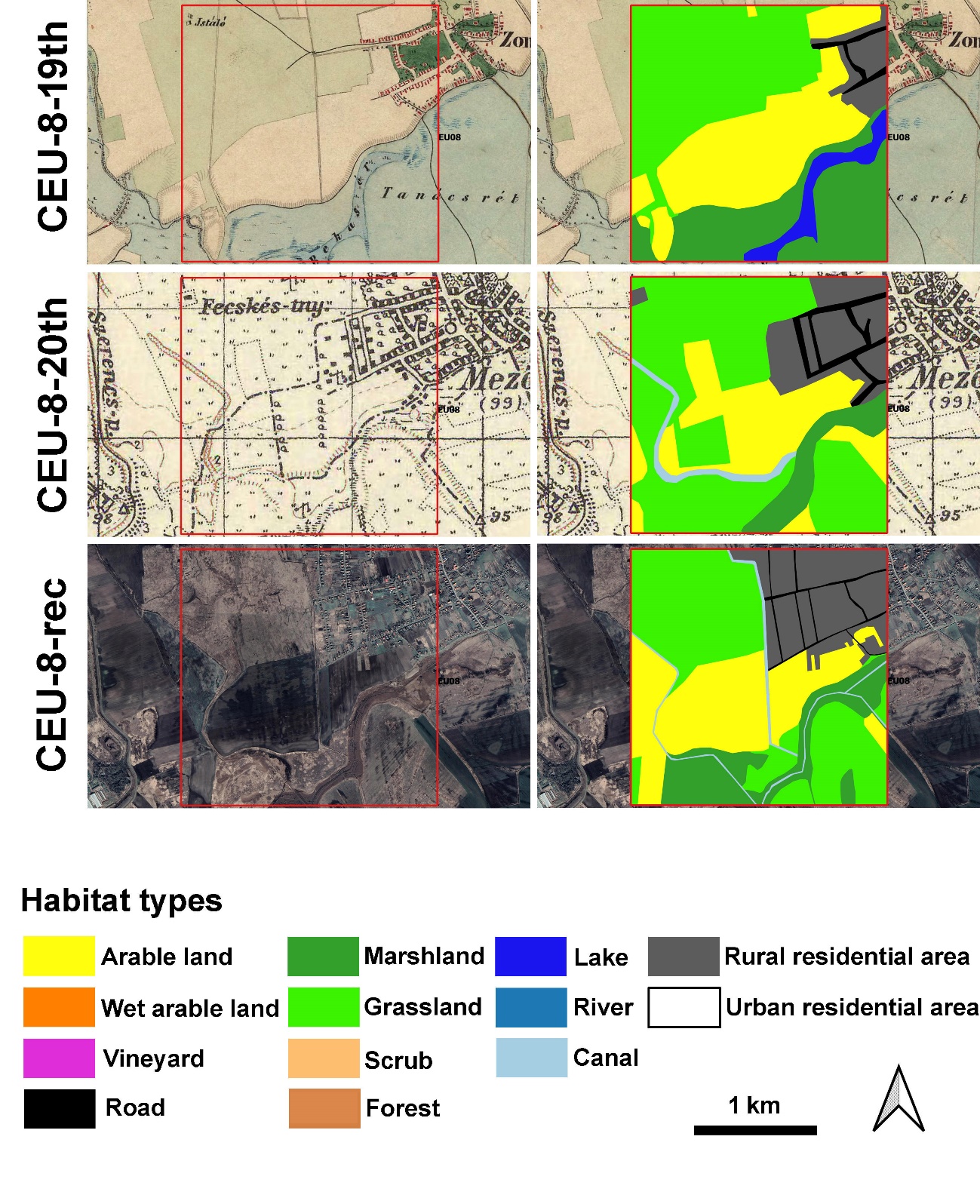


**Table S2h**. Relative surface cover of the recorded habitat types (abbreviations see in the manuscript), Malaria cases (MC) per 10,000 citizens, annual mean temperature (MT) and annual precipitation (AP) in CEU08 study site.

|  | **Ar** | **Ca** | **Fo** | **Gr** | **La** | **Ma** | **Ri** | **Ro** | **Ru** | **Sh** | **Ur** | **Wa** | **Wy** |
| --- | --- | --- | --- | --- | --- | --- | --- | --- | --- | --- | --- | --- | --- |
| **19^th^** | 0.298 | 0.000 | 0.000 | 0.347 | 0.047 | 0.244 | 0.000 | 0.012 | 0.052 | 0.000 | 0.000 | 0.000 | 0.000 |
| **20^th^** | 0.222 | 0.025 | 0.000 | 0.505 | 0.000 | 0.071 | 0.000 | 0.034 | 0.142 | 0.000 | 0.000 | 0.000 | 0.000 |
| **rec** | 0.284 | 0.023 | 0.000 | 0.354 | 0.000 | 0.137 | 0.000 | 0.016 | 0.187 | 0.000 | 0.000 | 0.000 | 0.000 |

|  | **MC** | **MT (°C)** | **AP (mm)** |
| --- | --- | --- | --- |
| **19^th^** | 185 | 13.1 | 673.9 |
| **20^th^** | 45 | 9.6 | 699.2 |
| **rec** | 0 | 12.8 | 676.9 |

**Figure S2i**. Habitat maps of the CEU09 study site.


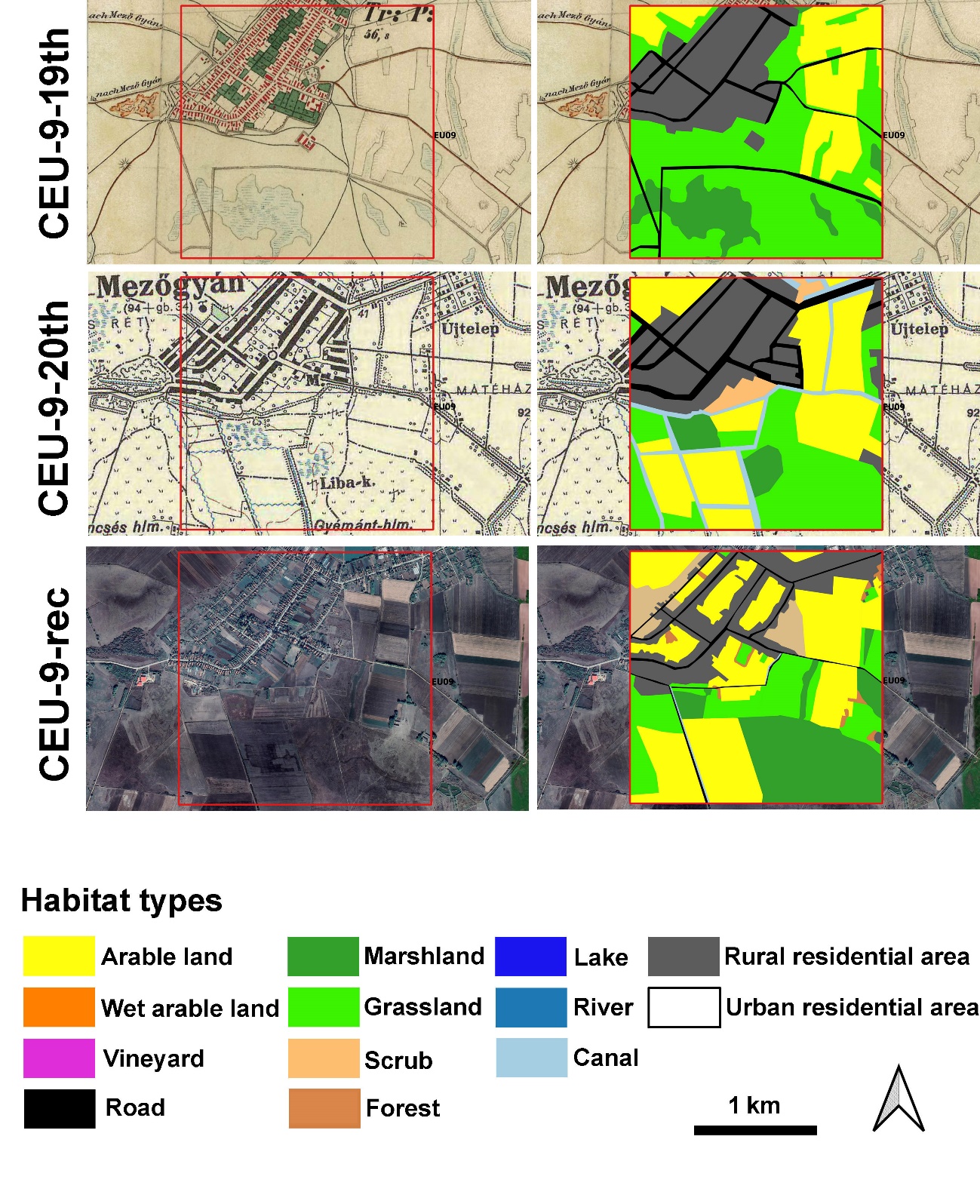


**Table S2i**. Relative surface cover of the recorded habitat types (abbreviations see in the manuscript), Malaria cases (MC) per 10,000 citizens, annual mean temperature (MT) and annual precipitation (AP) in CEU09 study site.

|  | **Ar** | **Ca** | **Fo** | **Gr** | **La** | **Ma** | **Ri** | **Ro** | **Ru** | **Sh** | **Ur** | **Wa** | **Wy** |
| --- | --- | --- | --- | --- | --- | --- | --- | --- | --- | --- | --- | --- | --- |
| **19^th^** | 0.178 | 0.000 | 0.000 | 0.464 | 0.000 | 0.092 | 0.000 | 0.060 | 0.205 | 0.000 | 0.000 | 0.000 | 0.000 |
| **20^th^** | 0.313 | 0.069 | 0.000 | 0.247 | 0.000 | 0.044 | 0.000 | 0.075 | 0.227 | 0.024 | 0.000 | 0.000 | 0.000 |
| **rec** | 0.381 | 0.004 | 0.011 | 0.150 | 0.000 | 0.190 | 0.000 | 0.030 | 0.184 | 0.050 | 0.000 | 0.000 | 0.000 |

|  | **MC** | **MT (°C)** | **AP (mm)** |
| --- | --- | --- | --- |
| **19^th^** | 75 | 11.2 | 614.4 |
| **20^th^** | 85 | 10.4 | 695.8 |
| **rec** | 0 | 13.2 | 661.0 |

**Figure S2j**. Habitat maps of the CEU10 study site.


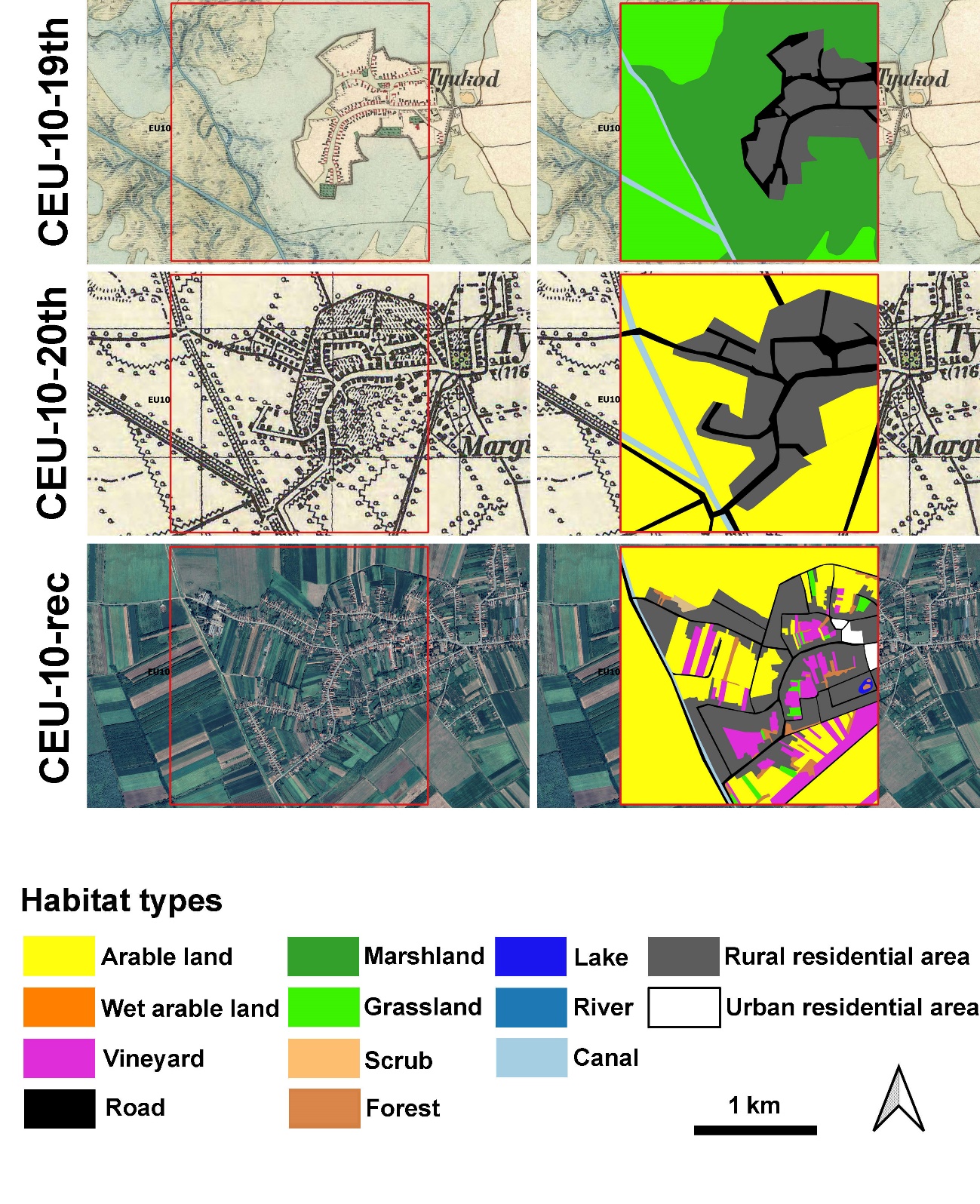


**Table S2j**. Relative surface cover of the recorded habitat types (abbreviations see in the manuscript), Malaria cases (MC) per 10,000 citizens, annual mean temperature (MT) and annual precipitation (AP) in CEU10 study site.

|  | **Ar** | **Ca** | **Fo** | **Gr** | **La** | **Ma** | **Ri** | **Ro** | **Ru** | **Sh** | **Ur** | **Wa** | **Wy** |
| --- | --- | --- | --- | --- | --- | --- | --- | --- | --- | --- | --- | --- | --- |
| **19^th^** | 0.000 | 0.021 | 0.000 | 0.293 | 0.000 | 0.486 | 0.000 | 0.048 | 0.153 | 0.000 | 0.000 | 0.000 | 0.000 |
| **20^th^** | 0.590 | 0.033 | 0.000 | 0.000 | 0.000 | 0.000 | 0.000 | 0.101 | 0.277 | 0.000 | 0.000 | 0.000 | 0.000 |
| **rec** | 0.533 | 0.010 | 0.022 | 0.012 | 0.002 | 0.000 | 0.000 | 0.051 | 0.263 | 0.003 | 0.010 | 0.000 | 0.094 |

|  | **MC** | **MT (°C)** | **AP (mm)** |
| --- | --- | --- | --- |
| **19^th^** | 195 | 11.5 | 663.9 |
| **20^th^** | 40 | 8.5 | 694.2 |
| **rec** | 0 | 12.8 | 678.5 |

**Figure S2k**. Habitat maps of the AF01, AF02, AF03, AF04 study sites.


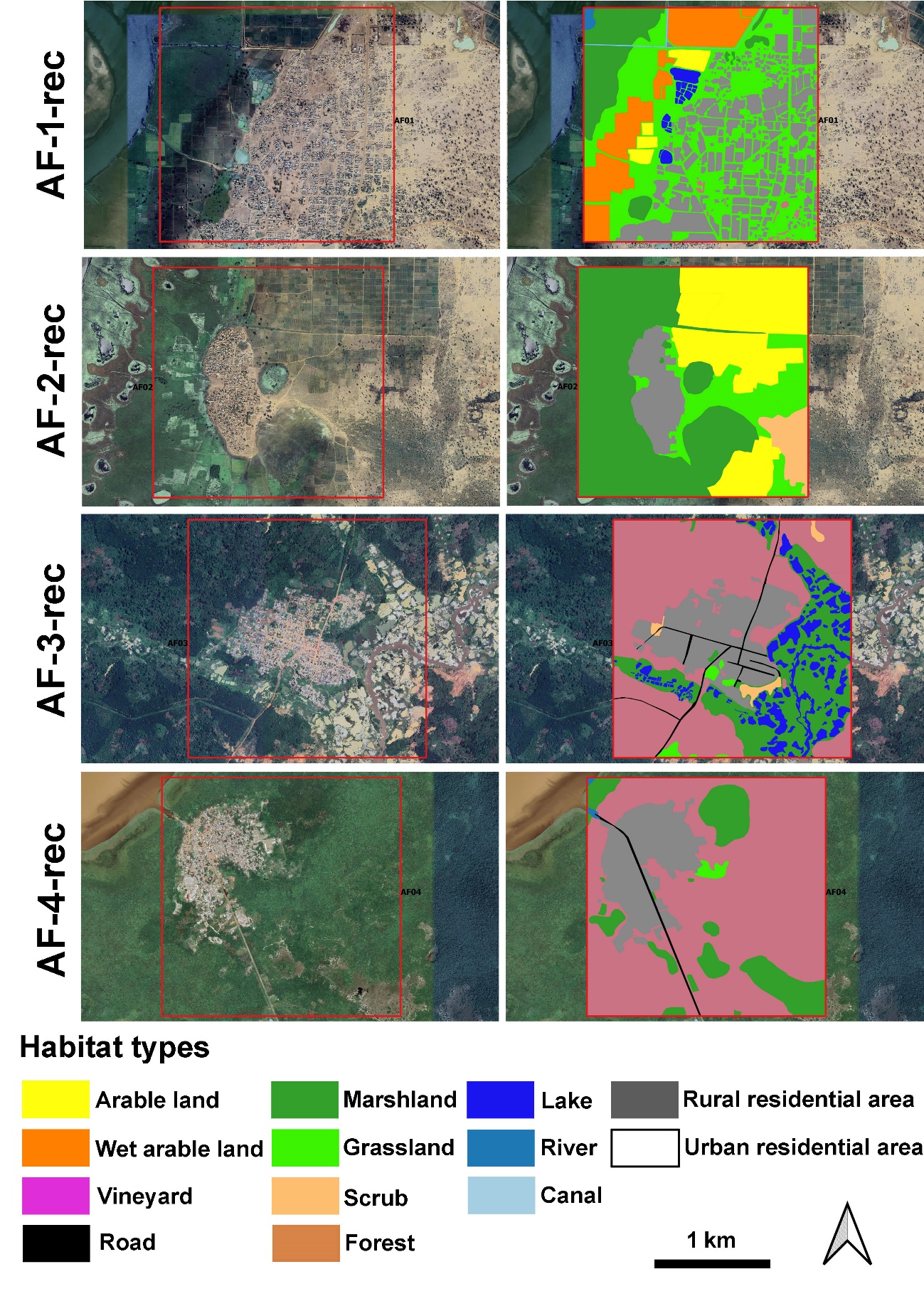


**Table S2k**. Relative surface cover of the recorded habitat types (abbreviations see in the manuscript) in AF01, AF02, AF03, AF04 study sites.

|  | **Ar** | **Ca** | **Fo** | **Gr** | **La** | **Ma** | **Ri** | **Ro** | **Ru** | **Sh** | **Ur** | **Wa** | **Wy** |
| --- | --- | --- | --- | --- | --- | --- | --- | --- | --- | --- | --- | --- | --- |
| **AF01** | 0.025 | 0.005 | 0.006 | 0.405 | 0.016 | 0.108 | 0.004 | 0.000 | 0.283 | 0.000 | 0.000 | 0.148 | 0.000 |
| **AF02** | 0.307 | 0.000 | 0.000 | 0.120 | 0.000 | 0.443 | 0.000 | 0.000 | 0.093 | 0.037 | 0.000 | 0.000 | 0.000 |
| **AF03** | 0.000 | 0.000 | 0.500 | 0.010 | 0.102 | 0.195 | 0.000 | 0.016 | 0.165 | 0.012 | 0.000 | 0.000 | 0.000 |
| **AF04** | 0.000 | 0.000 | 0.695 | 0.007 | 0.000 | 0.137 | 0.002 | 0.008 | 0.151 | 0.000 | 0.000 | 0.000 | 0.000 |

**Figure S2l**. Habitat maps of the AF05, AF06, AF07, AF08 study sites.


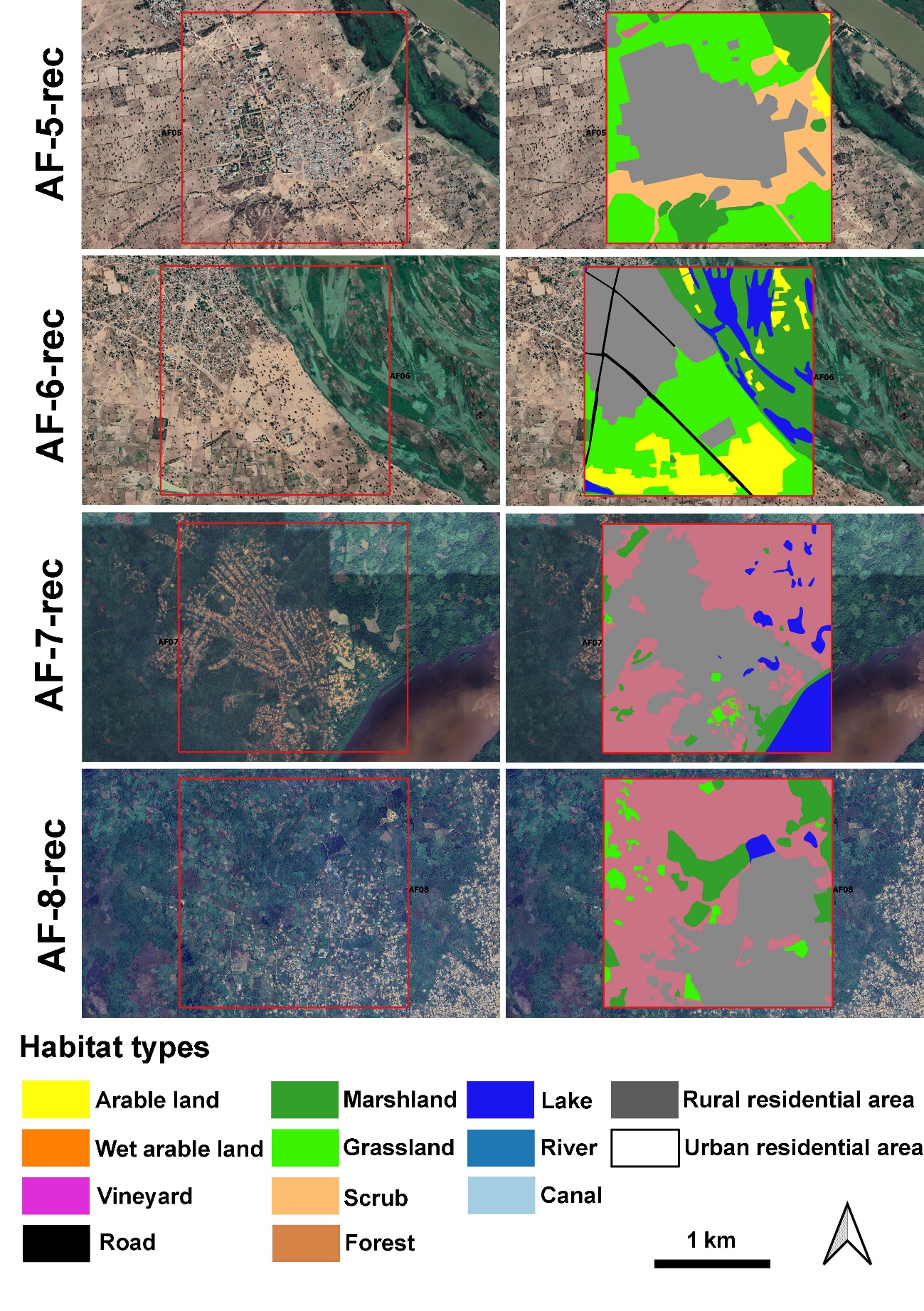


**Table S2l**. Relative surface cover of the recorded habitat types (abbreviations see in the manuscript) in AF05, AF06, AF07, AF08 study sites.

|  | **Ar** | **Ca** | **Fo** | **Gr** | **La** | **Ma** | **Ri** | **Ro** | **Ru** | **Sh** | **Ur** | **Wa** | **Wy** |
| --- | --- | --- | --- | --- | --- | --- | --- | --- | --- | --- | --- | --- | --- |
| **AF05** | 0.014 | 0.000 | 0.004 | 0.322 | 0.000 | 0.087 | 0.000 | 0.000 | 0.394 | 0.151 | 0.000 | 0.028 | 0.000 |
| **AF06** | 0.174 | 0.000 | 0.000 | 0.285 | 0.004 | 0.236 | 0.000 | 0.029 | 0.273 | 0.000 | 0.000 | 0.000 | 0.000 |
| **AF07** | 0.000 | 0.000 | 0.439 | 0.009 | 0.086 | 0.042 | 0.000 | 0.000 | 0.424 | 0.000 | 0.000 | 0.000 | 0.000 |
| **AF08** | 0.000 | 0.000 | 0.498 | 0.041 | 0.012 | 0.119 | 0.000 | 0.000 | 0.330 | 0.000 | 0.000 | 0.000 | 0.000 |

**Figure S2m**. Habitat maps of the AF09, AF10 study sites.


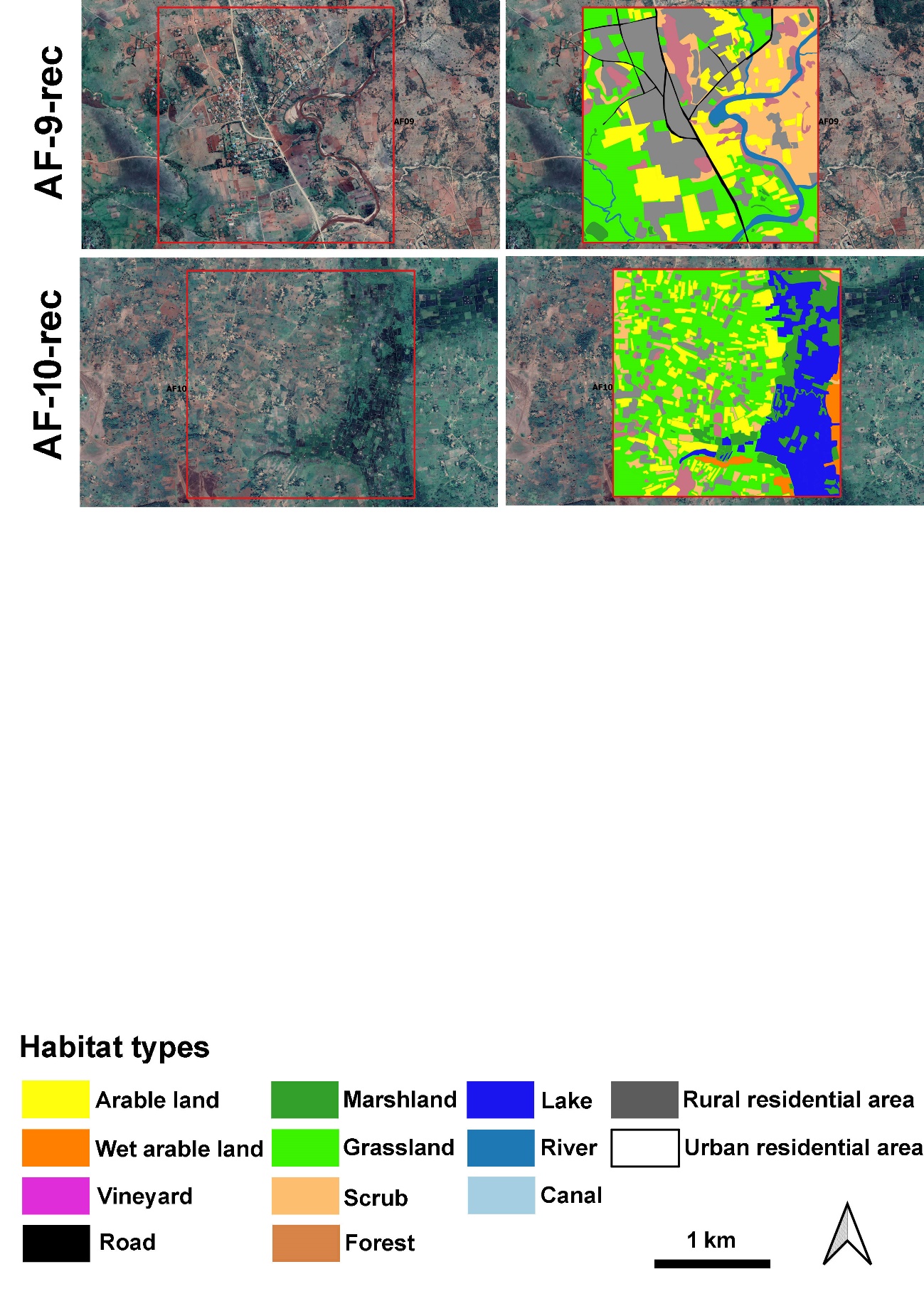


**Table S2m**. Relative surface cover of the recorded habitat types (abbreviations see in the manuscript) in AF09, AF10 study sites.

|  | **Ar** | **Ca** | **Fo** | **Gr** | **La** | **Ma** | **Ri** | **Ro** | **Ru** | **Sh** | **Ur** | **Wa** | **Wy** |
| --- | --- | --- | --- | --- | --- | --- | --- | --- | --- | --- | --- | --- | --- |
| **AF09** | 0.155 | 0.000 | 0.062 | 0.291 | 0.000 | 0.011 | 0.045 | 0.023 | 0.257 | 0.156 | 0.000 | 0.000 | 0.000 |
| **AF10** | 0.131 | 0.000 | 0.031 | 0.408 | 0.215 | 0.071 | 0.000 | 0.000 | 0.072 | 0.045 | 0.000 | 0.027 | 0.000 |

**Table S2n**. Malaria cases (MC) per 10,000 citizens, annual mean temperature (MT) and annual precipitation (AP) in the African study sites.

|  | **MC** | **MT (°C)** | **AP (mm)** |
| --- | --- | --- | --- |
| **AF01** | 400 | 27.2 | 720 |
| **AF02** | 450 | 27.4 | 748 |
| **AF03** | 6000 | 25.7 | 1348 |
| **AF04** | 5400 | 25.5 | 1300 |
| **AF05** | 4800 | 27.6 | 1220 |
| **AF06** | 4900 | 27.3 | 1309 |
| **AF07** | 2400 | 23.4 | 1543 |
| **AF08** | 2300 | 24.0 | 1380 |
| **AF09** | 50 | 19.8 | 1090 |
| **AF10** | 100 | 21.3 | 1180 |
